# Large-scale cortical travelling waves predict localized future cortical signals

**DOI:** 10.1371/journal.pcbi.1007316

**Published:** 2019-11-15

**Authors:** David M. Alexander, Tonio Ball, Andreas Schulze-Bonhage, Cees van Leeuwen

**Affiliations:** 1 Perceptual Dynamics Laboratory, Brain and Cognition Research Unit, KU Leuven, Leuven Belgium; 2 Medical Center - University of Freiburg, Translational Neurotechnology Lab, Freiburg i.Br., Germany; 3 Neurozentrum der Albert-Ludwigs-Universität, Sektion Epileptologie, Freiburg i.Br., Germany; Ludwig-Maximilians-Universitat Munchen, GERMANY

## Abstract

Predicting future brain signal is highly sought-after, yet difficult to achieve. To predict the future phase of cortical activity at localized ECoG and MEG recording sites, we exploit its predominant, large-scale, spatiotemporal dynamics. The dynamics are extracted from the brain signal through Fourier analysis and principal components analysis (PCA) only, and cast in a data model that predicts future signal at each site and frequency of interest. The dominant eigenvectors of the PCA that map the large-scale patterns of past cortical phase to future ones take the form of smoothly propagating waves over the entire measurement array. In ECoG data from 3 subjects and MEG data from 20 subjects collected during a self-initiated motor task, mean phase prediction errors were as low as 0.5 radians at local sites, surpassing state-of-the-art methods of within-time-series or event-related models. Prediction accuracy was highest in delta to beta bands, depending on the subject, was more accurate during episodes of high global power, but was not strongly dependent on the time-course of the task. Prediction results did not require past data from the to-be-predicted site. Rather, best accuracy depended on the availability in the model of long wavelength information. The utility of large-scale, low spatial frequency traveling waves in predicting future phase activity at local sites allows estimation of the error introduced by failing to account for irreducible trajectories in the activity dynamics.

## Author summary

Prediction is an important step in scientific progress, often leading to real-world applications. Prediction of future brain activity could lead to improvements in detecting driver and pilot error or real-time brain testing using transcranial magnetic stimulation. Previous studies have either supposed that the ‘noise’ level in the cortex is high, setting the prediction bar rather low; or used localized measurements to predict future activity, with modest success. A long-held but controversial hypothesis is that the cortex is best characterized as a multi-scale dynamic structure, in which the flow of activity at one scale, say, the area responsible for motor control, is inextricably tied to activity at smaller and larger scales, for example within a cortical column and the whole cortex. We test this hypothesis by analyzing large-scale traveling waves of cortical activity. Like waves arriving on a beach, the ongoing wave motion allows better prediction of future activity compared to monitoring the local rise and fall; in the best cases the future wave cycle is predicted with as low as 20° average error angle. The prediction techniques developed for the present research rely on mathematics related to quantifying large-scale weather patterns or analysis of fluid dynamics.

## Introduction

Predicting future cortical activity from previous measurements is notoriously hard. Yet, it is desirable for trial-by-trial experimental manipulations and constitutes the Holy Grail for brain computer interfaces, e.g., to anticipate critical changes in the brain state of neurological patients, drivers, pilots, or gamers, and for closed-loop transcranial magnetic stimulation [1]. Moreover, successful prediction of target signals would settle issues about signal quality under various measurement contingencies (e.g. extra- versus intra-cranial observation [2–4]). Because an artefactual account of the signal is unlikely to offer much predictive success, prediction is a more convincing criterion for signal veracity than description or correlation.

Extra- and intra-cranial measurements of cortical activity are typically considered noisy [5,6]; c.f. [7–9]. Most efforts to predict future activity have therefore been focused on eliminating noise through aggregation of data over time or trials. Such a focus delimits the scope of cortical signal prediction to statistical approaches, typified by Granger causality, as well as event-related approaches [7,10–12]. We have previously argued that aggregation, rather than removing noise, washes out non-additive portions of the signal. Doing so destroys, in particular, information about traveling wave (TW) patterns in cortical activity [3,13,14]. When these non-additive portions of the signal improve the prediction of future activity, this provides a clear-cut validation of their significance.

For electrocorticographic and encephalographic (ECoG and EEG) signals, so far the best predictions have been achieved using auto-regression [15] and the Fourier transform [16]. These methods make use solely of local information from the site at which a time-series is predicted, using past phase as a forward predictor of the phase in the near future. The prediction is compared to the actual phase by computing the phase-locking values of the phase errors. Zero prediction error in phase angle would yield a PLV_error_ of 1.0; random prediction would yield a PLV_error_ of 0.0. Generally, there is a high degree of variability in prediction success across subjects [1,15,16], which we also find in the present study. The previous literature has reported the case of the best predicted sensor, and selected trials according to power in the relevant band, which for the sake of comparison we also follow in the present work. The best subject’s results, using these criteria, yield PLV_error_ of 0.77 and 0.5 for EEG [16] and ECoG [15], respectively. These values correspond to average error angles of ~40° and ~60°, respectively.

Instead of local information, we use large-scale cortical dynamics in the form of TWs to predict future phase at localized recording sites. Prediction accuracy improves over the previous state-of-the art: ~20° average error angle for the best MEG subject, and ~30° for ECoG. The accuracy of TW methods is superior even if the prediction procedure disregards the past signal from the local to-be-predicted sensor site. Thus, large-scale spatiotemporal patterns allow better predictions of future local signals than can be obtained solely from past local signals. With this result, we envision to establish the non-artefactual nature of TW dynamics.

In addition, we show that the large-scale model performs comparably to event-related models. While the latter are intrinsically constrained by the requirements that (1) the nature and timing of the event are exactly known and (2) the subject’s interactions with the event are consistent across repeated trials, the present approach can be applied without these constraints to arbitrary data segments.

### The study of large-scale traveling waves in the cortex

Cortical TWs, along with standing waves, were first predicted from theoretical considerations more than three decades ago [17]. The proposed generation of these waves is derived from the electro-physiological properties of cortical cells and known fiber types; for example long-range cortico-cortical fibres in the case of large scale alpha waves [18–20]. Cortical TWs have been observed using optical imaging [21,22], LFP [23–27], ECoG [2–4], EEG [28,29,29–32] and MEG [3,13,33,34]. They play a prominent role in signals at multiple scales, ranging from columns [23,24], Brodmann areas [21,22,25–27,35], spanning several cortical areas [3,4,33,36,37,37] and whole cortex [28,29,33,36,38]. The latter, large-scale, TWs have been measured during sleep [38], rest [39] and during specific tasks [32]. For example, there are close correspondences between TW components (measured at the single trial level) and the latency topography of known visual and auditory ERP components, such as the P1–N1 complex, P2–N2 complex, as well as the P3b [28,31,40–42].

As mentioned, the standard view has been that widespread non-event-related signal can be regarded as *noise* relative to the *signal* of localized event-related activity [3,14]. We may question this view for three reasons. First, ongoing activity dominates the amplitude of the event-related signal by a factor of ten [6,7]. Second, large-scale patterns of activity dominate the smaller-scale patterns of activity in the cortex, since signals measured from the cortex have an approximately 1/f^m^ spatial spectrum [43,44], where the exponent *m* varies depending on whether the observations are intra- or extra-cranial. Together, these two findings imply that ongoing activity dominates the event-related signal everywhere in the cortex, not just at the experimenter-defined site of interest.

Third, large-scale cortical waves are observed in the ECoG/EEG/MEG and explain the major part (>50%) of the variance in phase patterns of the signal [13,28]. This class of waves have been reported at a variety of temporal frequencies, from the sub-delta through to gamma bands [2–4,28–30,33,38]. The three observations about the dominant activity of the cortex led us to suggest that TWs play an important role in relation to the localized, event-related activity, to wit: the localized, event-locked activity represents that portion of ongoing dynamics that arrive ‘just in time’ to the functionally relevant area for the execution of the experimental task [13]. Proof of this claim would be to show that the trajectory of the ongoing activity pattern can be used to improve the prediction accuracy of the locally measured signal; this is what is achieved in the present research.

The different filtering characteristics of ECoG and MEG allow us to assess the prediction efficacy of TWs in the approximate range 20 to 60cm wavelengths (10 to 30cm in sensor array coordinates). In MEG, the TW signal is dominated by long wavelengths [13], due primarily to the distance between the cortical activity and MEG coils. ECoG signal has an approximately 1/f^m^ spatial spectrum [18,43,45], and therefore the measured wavelengths more closely depend on the size of the measurement array used.

### Using large-scale dynamics to predict future phase

These features of large-scale cortical activity allow us to formalize the key theoretical argument driving the present research. Insofar as cortical signals behave as TWs, they are not space-time separable [12,13]. This means that the signal can only be partially captured by separate analyzes of their spatial extent and time-course. Almost all commonly used techniques in analysis of brain signals, including ERPs, coherence and Independent Components Analysis (ICA), implicitly assume space-time separability [13]. This assumption equates cortical activity *A* with the product of independent functions *F* of time, *t*, and *G* of space, *x*, as expressed in Eq 1:

A(t,x)=F(t)×G(x) (1)

In short, if a quantitative technique results in separate functions for the arguments *t* and *x*, then it assumes space-time separability. This assumption admittedly simplifies analysis [46], and has taught us a great deal about cortical functioning. It allows signals to be spatially smoothed, temporally convolved, temporally aggregated, spatially clustered and then spatially differenced to produce, for instance, standard fMRI difference images [3,47]. In the discussion, we describe the assumption of space-time separability in relation to methods of coherence, Beamformer filtering, event-related potentials and ICA.

Yet, as signals flow through the cortex over time in a variety of directions, precisely their characteristic trajectories will be lost when such aggregation procedures are applied, either across samples or trials [2,3]. As a result, the assumption of space-time separability distorts signal classification in a manner analogous to the barber pole illusion [48]. In the limiting case of single measurement site, it becomes impossible to distinguish between temporal frequency, spatial frequency and velocity effects; all variation across these dimensions is collapsed into the measured temporal frequency. Consider how this works out when, for instance, the discrete Fourier transform in space and time is used to analyze the activity:

$$\hat{f}\left(\omega ,\xi \right)=\sum_{t=0}^{N_{t}-1}\sum_{x=0}^{N_{x}-1}A\left[t,x\right]e^{-i\omega t-i\xi x}$$ (2)

where $\hat{f}$ is the Fourier component at temporal frequency *ω* and spatial frequency *ξ*, and *N*_*t*_ and *N*_*x*_ are the number of samples in time and space. The signal measurement site *j* can then be characterized at as

$${\hat{f}}_{x=j}(\omega ,\xi )=\sum_{n_{t}=0}^{N_{t}-1}F[t]e^{-i\omega t}\cdot \varepsilon (\xi )$$ (3)

where the final term, *ε*(*ξ*), is the error introduced by ignoring the spatio-temporal interactions of the signal. In most standard analyses (e.g. cross-trial coherence), *ε*(*ξ*) is considered a noise term, to be reduced by aggregation over times. But suppose function *A* contains an irreducible space-time interaction term. If so, predictive models assuming space-time separability will perform worse than ones that do not. The discrepancy between them can then be used to estimate *ε*(*ξ*). In the present research we focus on the error phase angle, ∠*ε*(*ξ*).

We expect non-trivial ∠*ε*(*ξ*) to arise when there is more than one mode of *A*, that is, if the distribution of spatio-temporal Fourier components does not have a single, narrow peak. In a system with only a single parameterization of the wave, using *F*(*t*) or *G*(*x*) to quantify wave behaviour would introduce no error in predicted phase angle. In this case there are no additional degrees of freedom in *A*(*t*,*x*), so no *irreducible* space-time *interaction* term. Under the assumption of a single mode, a topography of phase coherence would be sufficient to capture the space-time trajectory, as described in [36]. However, consider the more realistic case where the signal over time is constituted by families of waves travelling in different directions or at different speeds [13,28]. Now, quantifying the signal in terms of *F*(*t*) or *G*(*x*) fails to capture real variation in *A*(*t*,*x*) since the Fourier components reside in a higher dimensional space. This is the significance of Eqs 2 and 3, whereby limiting the analysis to *F*(*t*) introduces the error term *ε*(*ξ*). For similar reasons, event-related models that assume space-time separability will also introduce inaccuracies if there are multiple modes to *A*(*t*,*x*). We have previously argued [14] that this is akin to confusing the dominant frequencies of a Moiré pattern with those of its component images.

We take the approach of predicting future phase angle to assess the validity of space-time separability assumptions. Instead of assuming that the signal can be captured by modelling *F*(*t*) while holding *G*(*x*) constant (or vice versa), we propose to quantify *A*(*t*,*x*) directly. To achieve this, we empirically construct the Fourier components of the higher dimensional spatio-temporal system. This allows us to compare accuracy against a purely temporal Fourier model and hence to quantify any error angle, ∠ε(ξ), introduced by assuming space-time separability.

The first step in this procedure is estimation of phase angle from the raw time-series. Phase angle only has meaning for broad-band signals when the phase is estimated from a time-window equivalent to at least one cycle at the frequency of interest. In this sense the term ‘instantaneous phase’, in the context of brain signals, is a misnomer; there is always the initial step of estimating the narrow-band signals. The practical consequence of the definition of phase is that estimates of phase at adjacent samples will have a high degree of overlap in the raw signal used to estimate them; whether the estimation is achieved via band-pass filtering or finite impulse response filter, then the Hilbert transform, or directly via Morlet wavelets, as in the present research.

Intuitively, waves will predict phase at a nearby site in the near future in the direction of travel. However, because of the way phase is calculated, such predictions are trivial, unless we consider a minimum separation between past and predicted future phase. Specifically, the number of cycles of raw signal used to estimate phase is equivalent to the minimum time delay (at the frequency of interest) at which two successive estimates of phase are fully independent of each other. For example, two-cycle Morlet wavelets used to estimate the phase at 10Hz require a minimum temporal gap between past and future phase estimates of 200ms to avoid overlap between the raw signals used in the two estimates. Fortunately, as we will observe, traveling waves allow accurate prediction of future phase over at least one to two cycles.

### Overview of methods

**Fig 1:**
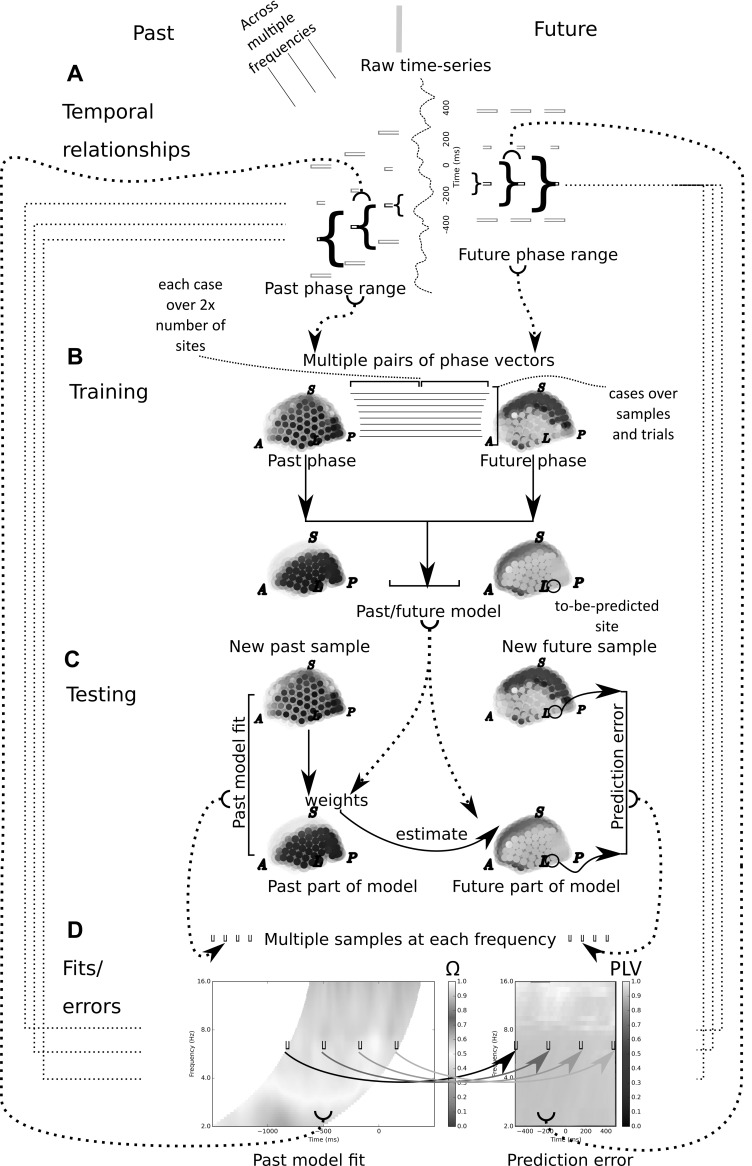
Schematic of analysis workflow A. Temporal relationships between predictor and predicted signal. The raw time-series is first analyzed using short-time-window Morlet wavelets to estimate the phase. The predictor and predicted phase are estimated from non-overlapping portions of the raw time-series. Since each phase estimate requires a window of the raw time-series (brackets), and this window is larger at low frequencies, this means the minimum temporal delay (milliseconds) between past and future phase estimates increases with decreasing frequency. The temporal delay was one cycle at the frequency of interest for the ECoG analysis, and two cycles for the MEG. B. Training stage. Principal components analysis (PCA) is used to empirically derive the spatio-temporal Fourier components. For a single subject, and for a single frequency, all the pairs past-future phase vectors available over all training trials were entered as cases into the PCA. The eigenvectors produced by the PCA were used as basis functions in the subsequent modelling. Each basis function was comprised of a past and future representation of the signal, by virtue of the structure of the paired input vectors. The site to be predicted was also chosen during the training stage, by finding the best predicted site from the future part of the model. For some analyzes, the training stage was performed again from scratch, save with the to-be-predicted site now omitted from the past part of the training vector. The most anterior site, most posterior, most superior and left-most site, are indicated by the letters A, P, S and L, respectively. C. Testing stage. A new past sample of phase (over all measurement sites, or possibly excluding the to-be-predicted site) from the test data set is used to estimate a set of model weights via regression onto the past part of the basis functions. These weights are then used to create a model representation of the future activity. The model phase at the to-be-predicted site is then compared to the actual phase that occurs at that site, forward in time. D. Fits of the past model and errors at the to-be-predicted future site. Left panel shows the fit of the past model to the past samples, averaged at each sample-within-trial; over trials (see Methods). The trial-averaged prediction error, for each sample, at the to-be-predicted future site is shown in the right panel (see methods). The critical feature to note is that each past sample is offset from its paired future sample by a delay that decreases with frequency, due to the requirement that phase be estimated from non-overlapping regions of the raw signal. Four equally spaced time-samples are indicated by ‘bins’ within the plot of past model fits, and their corresponding paired four future samples are likewise indicated within the plot of prediction errors. The paired samples have a corresponding representation in A, as the middle of the three frequencies shown. The relationship between frequency in the output plots (D) and the phase estimation windows for the raw time-series (A, brackets) is indicated by fine dashed lines. The second of the four paired past/future samples, at the middle frequency shown in A, is indicated by its position in the two output plots (D) via the half-circles brackets joined by bold dotted lines.

We construct a model of cortical activity to predict from each time in the trial (*past*) to another time in the trial (*future*), illustrated schematically in Fig 1. The model predicted the *future* phase at each particular frequency of interest. Two data sets were used in the present study, one set of MEG recordings [49] and one set of ECoG recordings [50]. The tasks were related, in that both involved a simple voluntary hand movement with either the left or the right hand. The stages of the computational procedure are illustrated in schematic form in Fig 1. First, the phase estimates were computed from the raw time-series using very short time-series Morlet wavelets. This was done for a large range of finely spaced frequency bands between 2 and 16Hz. Second, each sample of phase, over all the measurement sites, was paired with another sample of phase offset in time. The pairs were called the *past* and *future* samples. The time-offset between *past* and *future* samples was determined by the minimum delay to ensure that the two sets of phase estimates were from non-overlapping regions of the raw data. This means that for a given frequency, each *past* sample was matched with a *future* sample offset by a constant, frequency-dependent time shift. Third, the paired phase vectors for a single frequency were entered as cases in a principal components analysis (PCA). The eigenvectors from the PCA that explained the most variance were used as low dimensional representations (basis functions) to model the relationship between *past* and future *phase* vectors. Fourth, *past* phase samples were used to estimate model weights from the *past* part of the model bases. These weights were then used to construct a model of *future* activity by applying the weights to the *future* part of the model bases. Fifth, the model *future* activity was compared to the actual future activity that occurred for the relevant *future* sample. By comparing the prediction errors across all sites, the site that was best predicted at this frequency was found. Sixth, using the previously obtained set of basis functions, previously unseen trials were used to estimate the prediction accuracy of the model at the to-be-predicted site. The result is a prediction from each time in the trial (*past*) to another time in the trial (*future*).

## Results

We analyzed both MEG and ECoG data involving tasks that required subjects to initiate a simple movement with one hand or the other—either a button press with the index finger, in the case of the MEG data [49], or pressing index finger to thumb, in the case of the ECoG data [3,50]. We show that non-local phase patterns over large-scale cortex enable accurate prediction of future phase at individual sites. Our goal here was not to deploy a host of machine learning techniques to minimize prediction error, *per se*. Rather, our aim was to build a model based on the predominant characteristics of the large-scale cortical dynamics, based on previous theory of cortex [14,17,18]. For this reason, we extracted traveling wave information using Fourier analysis and Principal Component Analysis (PCA). Our approach is related to empirical orthogonal functions, used extensively in meteorology, oceanography and geophysics [51]. Our approach is also similar to dynamic mode decomposition [52], often used in fluid dynamics, but rather than estimating the system difference equations we model the relationship between past and future events.

### Empirical basis functions from PCA

Using PCA on patterns of phase has shown that more than half the variance in the spatial pattern of phase is explained by large scale (low spatial frequency, 10 to 30 cm in sensor coordinates) gradients of phase (2,23). The present research adds one critical dimension to this method of analysis: *past* and *future* patterns of phase are included in the same model. The best predicted site is determined during model construction, and previously unseen data is then used to assess prediction accuracy. We only report the best predicted site, allowing us to compare our results with previous expositions of cortical phase prediction techniques [15,16]. The model relates the past whole array dynamics, to the selected site’s future activity, via the future whole array dynamics.

**Fig 2:**
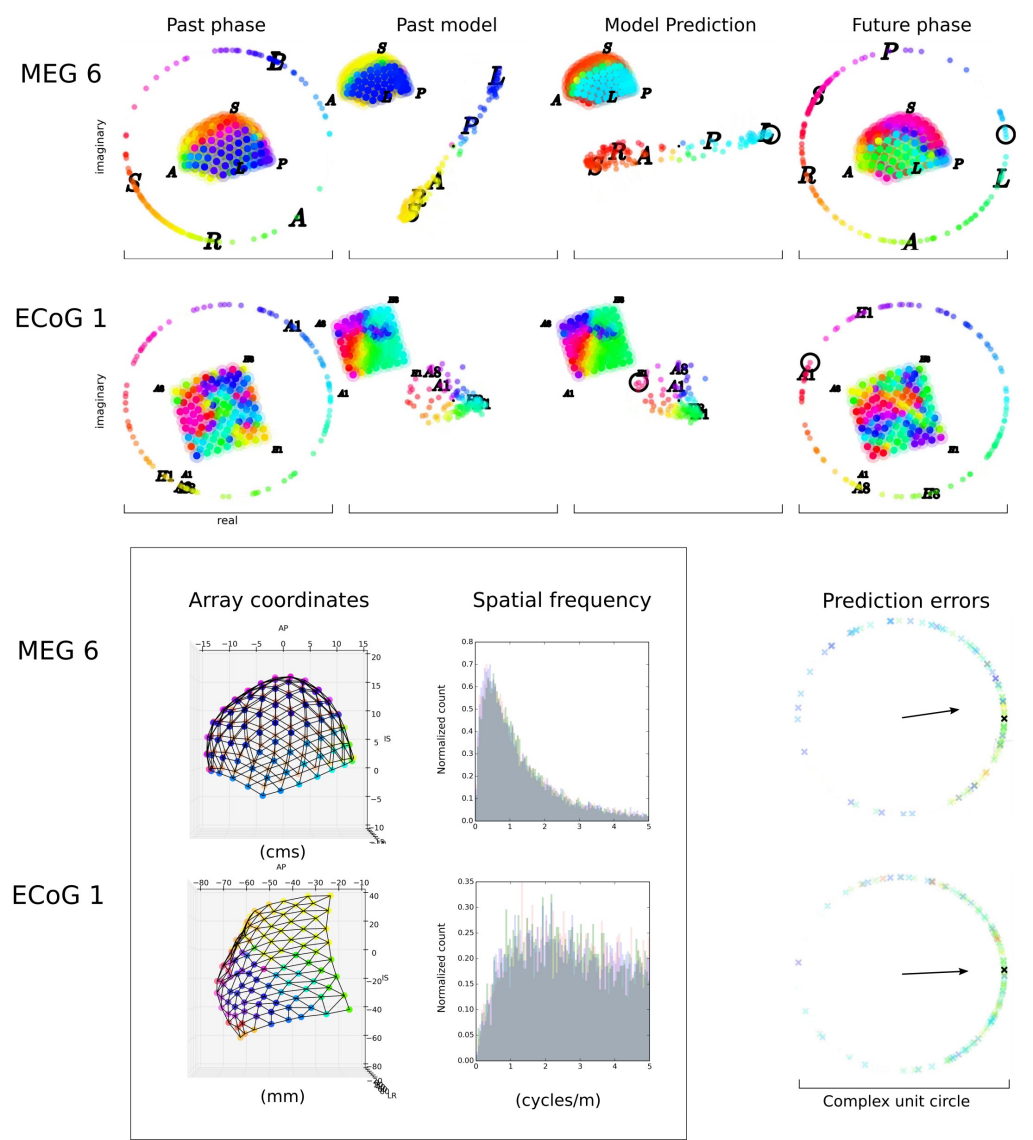
Example predictive models and prediction errors. The inset figure has two panels. The first panel gives the relative size and spacing of the measurement arrays for two subjects. The three axes are ‘AP’: anterior-posterior; ‘IS’: inferior-superior; ‘LR’: left-right. A typical phase gradient is shown in colour from the scalar values of phase. The second panel shows the distributions of estimated spatial frequency of the measured phase, over all trials and samples for the subject. The distribution of estimated spatial frequency of phase gradients (see Methods) is shown for all samples in grey. The figure is composed by overlay of samples drawn from the top third of prediction accuracy (red), middle third (green), and lower third (blue). Since selection by prediction accuracy does not alter the distribution, the combined plot is mostly grey. The main figure is broken into five panels. The first four panels show the past phase (as a head map and on the complex plane), the past model phase, the model prediction of the future phase, the actual future phase. For the past phase and future phase panels, head maps are shown in the centre of the panel, with unit circle of phase surrounding. The colours are used to represent phase in both head and circle, and phase is reiterated as angular position on the unit phase circle. For the past model and model prediction, the model output is shown on the complex plane in the centre of the panel. The argument of the model phase i.e. the angle, is shown in colours here and on the model head map (top left of these two panels). The site to be predicted is indicated with a black circle in both the model prediction panel and the future phase panel. These two example predictions are built using only the first eigenvector from the PCA, and therefore the second and third panels illustrate that eigenvector. Upper row: Subject MEG 6, centre frequency 2.0Hz, to-be-predicted site left out of past model. The letters ‘A’, ‘P’, ‘I’, ‘S’, ‘L’, ‘R’ indicate the position of the anterior-most (posterior-most etc.) recording site on either the head map or the unit circle of phase. The site to be predicted is missing from the past model representation, on the bottom row of sensors, just to the right of the ‘L’ sensor. This snapshot is taken during the third trial, at 364ms. See S1 Video, for the first three trials of this subject. Lower row: Subject ECoG 1, centre frequency 6.7Hz. The letters ‘A1’, ‘A8’, ‘H1’, ‘H8’ indicate the labels and positions of the corner recording sites of the ECoG array. The site to be predicted is missing from the past model representation, in position ‘B6’ i.e. one column in from the left, three rows down. This snapshot is taken during the first trial, at -250ms. See S2 Video, for the first three trials of this subject. In the fifth panel (lower right of figure), the mean prediction error is shown with a black arrow for Subject MEG 6 at 354ms, 2.0Hz (upper) and Subject ECoG 1 at -250ms, 6.7Hz (lower). The prediction errors are for a specific site, chosen as the to-be-predicted site during the model construction phase. The phase locking value (PLV) is the real part (i.e. the length of this arrow along the x-axis), here, 0.58 and 0.64 respectively. The prediction errors for each individual trial are also shown with coloured crosses, hot colours are trials with high mean log power, cold colours with low. The prediction error for the trial shown in Fig 1 is indicated with a black cross.

As in previous reports [2,12,23], the first three (ECoG) or four (MEG) eigenvectors consisted of low spatial frequency patterns with wave number approximately unity over the sensor array. Examples of eigenvectors are shown in Fig 2, where the model shown used only the first eigenvector. The structure of the eigenvectors can be seen as smoothly changing patterns of phase in the *past* model and model prediction. Across subjects, the low spatial frequency bases explained 20% to 53% of the variance in phase, lower than when only a single sample of phase is input into the PCA [3,28]. Subsequent eigenvectors, after the first three or four, were spatial frequency doubled and then quadrupled, as reported previously [2].

The dominance of low spatial frequencies in the past/future bases is therefore consistent with the three observations about cortical dynamics outlined in the introduction. As further confirmation, we estimated the spatial frequency distributions of the local phase gradients for each subject. For the MEG subjects, typical estimated spatial frequency had a peak in the distribution between 0.5 and 1.5 cycles per metre, with a long tail for higher spatial frequencies. These estimates can be compared with estimates for the same MEG data using multi-grid techniques to fit the waves (~3 cycles per metre [13]). The present estimates, read from the peak of the local gradient distribution, give somewhat lower spatial frequencies, so the mean or median of the distribution likely gives a better match to the previous report. However, the present estimation procedures are only included to provide a rough indicator, so we merely note that they likely underestimate the spatial frequency. For ECoG subjects, the local gradient distribution had a similar peaked shape, but was peaked at around 2, 3 or 4 cycles per metre. Fig 2 shows sensor spacing and the estimated spatial frequency distributions for MEG Subject 6 and ECoG Subject 1. Subjects did not appear to differ in spatial frequency distributions when these were compared across prediction accuracy tertiles (red, green, blue colours).

The predictive models and their predictions are illustrated in Fig 2 (and S1 Fig). From the upper panels (MEG Subject 6) it can been seen that the pattern of predicted phase (‘Model Prediction’) matched the measured pattern of phase (‘Future Phase’); except the former is smoother. In ECoG Subject 1, the match is less obvious, although examination of S2 Video reveals many epochs where the match was accurate. The best predicted site has high magnitude values in the model i.e. *M*_*future*_ (see Methods, Supplementary Videos), as seen by the black circle in model prediction panels of Fig 2. In general, the models reduce the complicated spatial pattern of phase over the measurement array to a simpler, smoothed spatial gradient of phase. The gradient comprises an empirically constructed spherical Fourier decomposition of the spatio-temporal phase patterns (see S1 Methods).

The eigenvectors, and thus the waves in the models established in the construction stage, tended to be spiral patterns. This is due to the complex-valued phase representation; previous observations were that PCA produces linear TWs from scalar-represented (spatially-unwrapped) phase [13,28]. This difference is due to the constraint on the PCA, as a linear method, to express the cases as weighted sums of the eigenvectors. For complex-valued phase, a globally coherent spiral wave on a sphere, with decreasing magnitude phase vectors toward the rotation singularity, can be expressed as a weighted sum of three similarly structured, orthogonal, spiral wave bases. Likewise an arbitrary linear gradient traversing the surface of a sphere can be described as the weighted sum of three, orthogonal, scalar valued linear gradients of phase. We are currently exploring methods to combine the two representations into a unified cortical Fourier analysis method.

The model waves produced from the weighted eigenvectors sometimes differed in spatial topography for the *past* and *future* portions of the model, but usually were similar (see Supplementary Videos). In addition, the phase offset between the past and future patterns was not always equal to a multiple of 2π, despite the temporal delay between past and future samples being 2π/*f* (ECoG) or 4π/*f* (MEG), where *f* is the temporal frequency. Notably, this discrepancy runs counter to the assumption of a constant ongoing phase; as predicting future phase from single time-series would require [14,15], and contributes to the error term, ∠*ε*(*ξ*), described in Eq 3. Temporal frequency alone and spatio-temporal frequency give different estimates of the change in phase; as they should if the spatio-temporal Fourier components improve prediction accuracy (Eq 2) beyond a purely temporal Fourier model (Eq 3).

**Fig 3:**
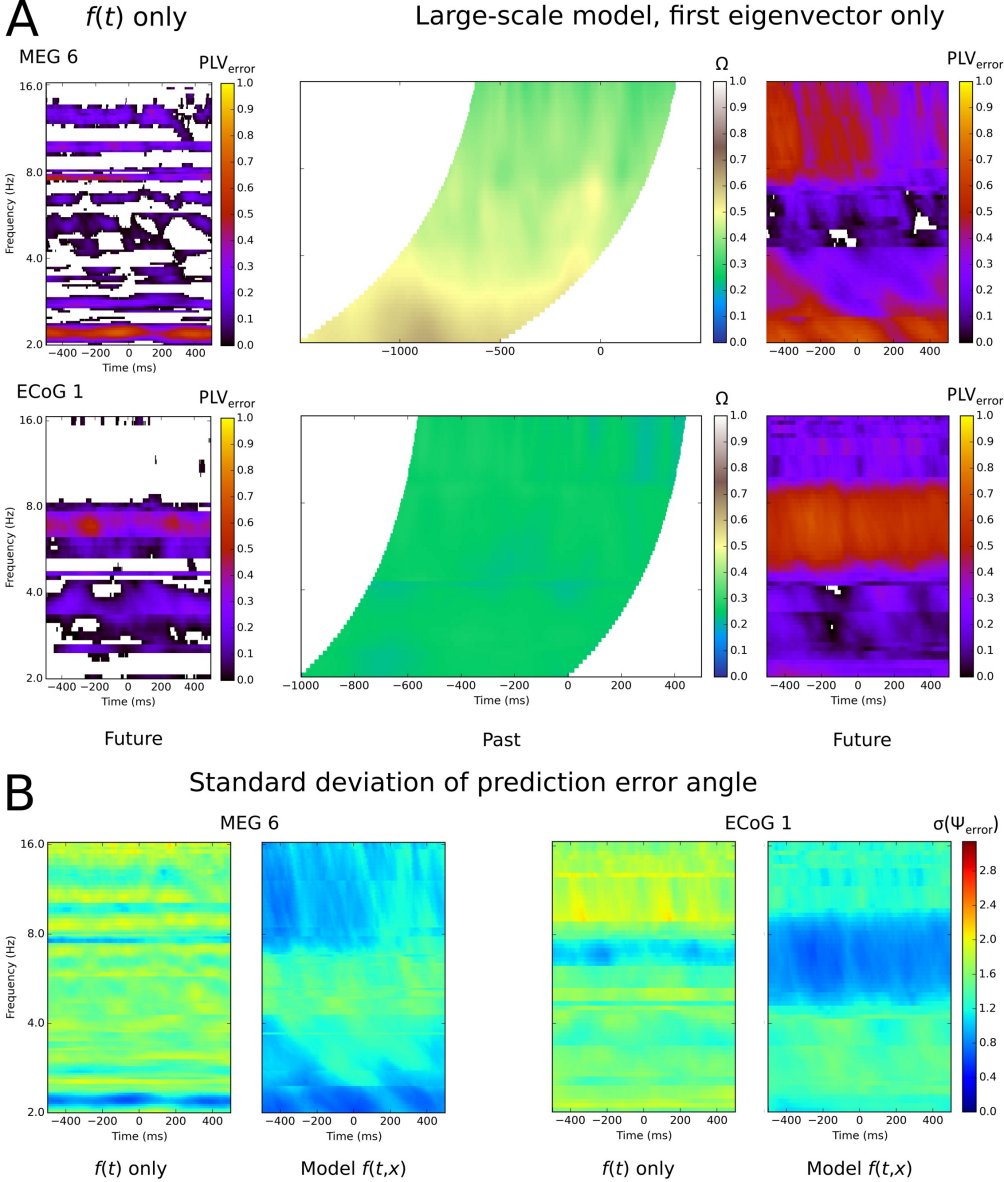
Temporal Fourier model and large-scale model results for two subjects over all samples and frequencies, single eigenvector only. A. The left plots show the PLV_error_ for the model that used only the temporal information at the site to be predicted. All values are from the test data set, for the same site analyzed in the middle and right panels. Middle plots show the fit of the past phase data to past model, Ω, the right plot shows the PLV_error_ for the best predicted site, chosen during model construction. All values are from the test data set. Both Ω and PLV_error_ extend over 1 second of data, and each sample in the past plot corresponds to the prediction error of the same sample in the future plot. The past plot samples are offset backwards time by either one (ECoG) or two (MEG) cycles at the frequency of interest, indicated by the curved boundaries. Blank regions within the future plot indicate PLV_error_ less than zero. B. The same future prediction errors as (A), expressed as the standard deviation of the error angle. Colour scale is in radians. The time-only Fourier model results are shown in the first and third panels, the large-scale spatio-temporal Fourier model results are shown in the second and fourth panels.

This effect is also implied by the results shown in Fig 3B. Here we compared, for two subjects, the average error angle for a purely temporal Fourier model and large-scale spatio-temporal Fourier model. Essentially, the purely temporal Fourier model gives somewhat higher error angles at the times and frequencies of best predictions. More striking, the range of temporal frequencies at which good predictions can be found is rather narrow for the purely temporal model, compared to spatio-temporal model. We interpret this effect as a broad peak(s) in the Fourier power spectrum $\hat{f}(\omega ,\xi )$ which is largely obscured by the considering only the slice at ${\hat{f}}_{x=j}(\omega )$. Note, however, that we are not measuring the power spectra, *per se*, but using the Fourier components to predict activity offset in the future.

### Large-scale model results

In the prediction stage of the analysis, the spatial pattern of phase for the *past* data was used to generate the predicted spatial gradient of phase for the *future* data. The predicted site’s value was then compared to the actual *future* phase value. The prediction error was calculated as the (real part of the) phase locking value (PLV), over all trials, of complex valued error vector at each sample within the trial’s time-course, as shown in Fig 2. The prediction errors per trial are shown as crosses on the unit circle, and are clustered in the direction of zero error (3 o’clock). The overall prediction accuracy is shown by the black arrow, which represents the average of the prediction errors. Only the length of the arrow in the horizontal direction is important to the assessment of prediction error, since the arrow represents the difference in model angle and angle to-be-predicted.

There was a wide variation between subjects in the success of the prediction procedure, as high as 0.73 PLV_error_ in some, and as low as 0.32 in others. Similar inter-subject variation has been reported elsewhere [14,15]. S2 Fig shows the relationship between PLV_error_ and average error angle, assuming a Gaussian distribution of errors, with a PLV_error_ of 0.7 corresponding to an average error angle of less than 60°. We found that 1-cycle Morlet wavelets performed best with the ECoG data, whereas 2-cycle Morlet wavelets performed best for the MEG data. Future research will explore whether this finding is general and what the reasons for it might be. Fig 3A shows time by frequency plots of model performance for the best subjects. The figure shows the fit of the *past* part of the model, Ω, which was greater in the lower frequency bands for MEG Subject 6 but was relatively uniform over times and frequencies for ECoG Subject 1. The prediction accuracy, for the to-be-predicted site, is shown in the right panel. The MEG subject’s future activity was predicted well in the delta and the alpha-beta range, but not in theta. The obverse was true for the ECoG subject. Generally speaking, the best PLV_error_s occurred in any of the beta, alpha, theta or delta bands depending on the subject. The PLV_error_ for the purely temporal Fourier model is provided in Fig 3A for comparison purposes, for the same predicted site as the large-scale model in each case.

To exclude dependency of the model’s predictive success on the number of eigenvectors used, we constructed models using incremental numbers of eigenvectors, starting from one. These variations showed similar relative performance over all frequencies. This result means that the first eigenvector of the PCA was sufficient to get nearly maximal prediction accuracy.

**Fig 4:**
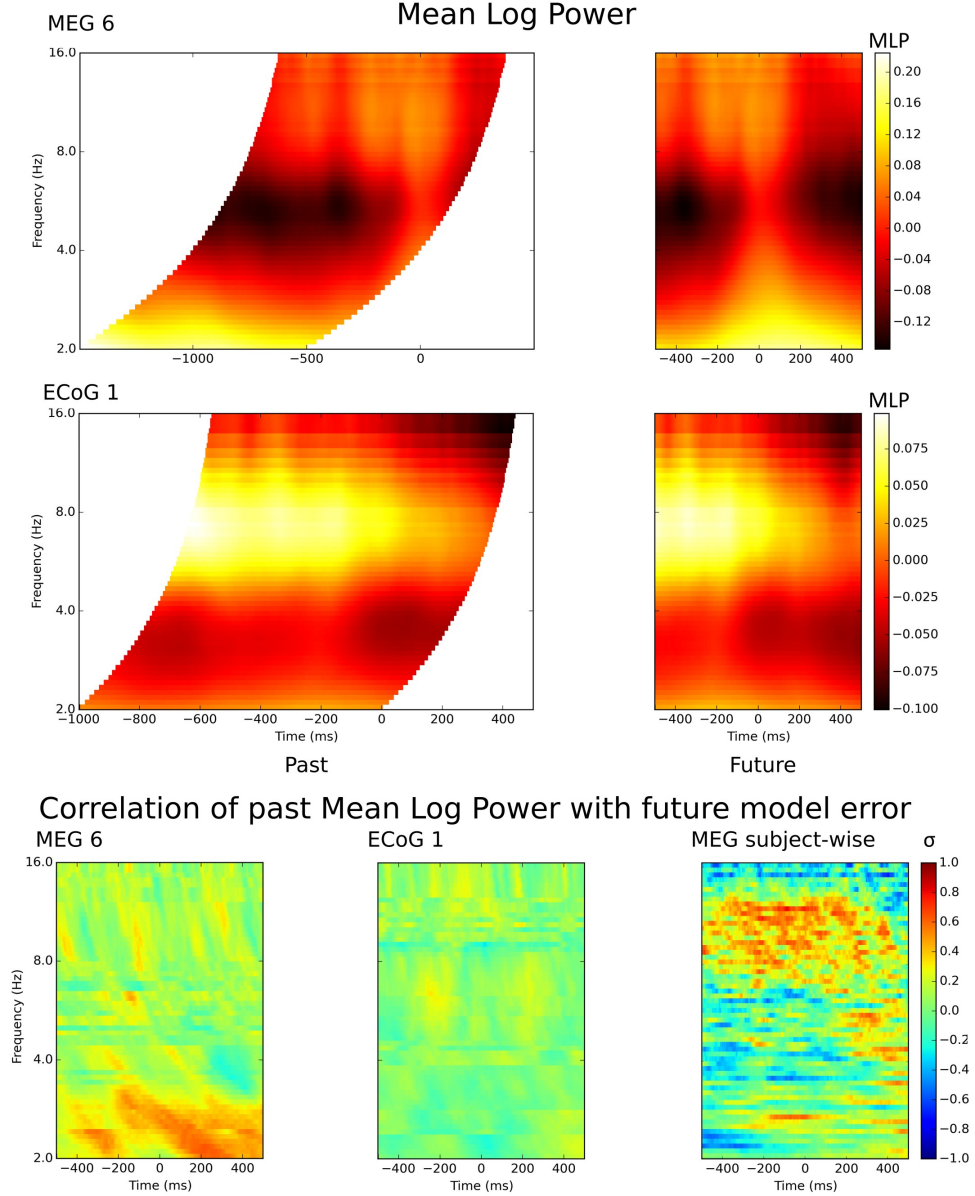
Mean log power and its relationship to model prediction error. Upper plots show MLP for the same two subjects in previous figures. MLP is normalized over the frequency range by subtracting a linear ramp as a function of frequency, so that event-related changes are emphasized rather than the 1/*f* power gradient. Conventions are otherwise the same as Fig 3. Lower plots show the time by frequency matrix of correlations between *past* MLP and prediction error. The real part of the trial-wise error vector (plots one and two) was generally correlated positively with the trial-wise *past* MLP. The PLV_error_ (plot three) was generally positively correlated with subject-wise *past* MLP.

For comparison purposes, in Fig 4 we include plots of mean log power (MLP) for our example subjects (see Methods). These plots help convey the temporal relationship between the *past* and *future* time-series. For Subject MEG 6, the landmark of a ‘waist’ of higher power can be seen across all frequencies, centred on time zero, in *past* and *future* plots, while the temporal range of the *past* time-series moves forward in time as the frequency increases. To be clear, only equivalent points in *past* and *future* temporal ranges were related to each other in the model e.g. the phase estimates at -200*ms* was used to predict the phase at 0*ms* for the 10*Hz* model in MEG, with phase estimated using two cycle Morlet wavelets.

We found that, for some frequencies and most subjects, there was a moderate correlation (*r*=0.3-0.6) between PLV_error_ and mean log power (see Fig 4). The correlation was widespread in time, for several hundred milliseconds either side of the button press response. For MEG Subject 6, the strongest correlations were late in the trial, in the delta band (*n*=126; *p*<0.05 at |*r*|>0.175). For ECoG Subject 1, the correlations were weaker but more widespread (*n*=150; *p*<0.05 at |*r*|>0.161). Consistent with these single subject observations, the subjects that had highest PLV_error_ also had high power in similar frequency bands, revealed as moderate between-subject correlations between PLV_error_ and mean power (MEG *n*=20; *p*<0.05 at |*r*|>0.445). This occurred chiefly in the alpha band, and later in the trial also in the delta band, where high PLV_error_s also tended to be found.

By taking into account the *past* power values during the testing step, we were able to improve the prediction performance. When only the highest 25% of trials, by *past* MLP, were included, the performance of the model improved to PLV_error_=0.94 for the best subject, with mean improvement over subjects of 0.17 (see Table 1). This best PLV_error_ corresponds to a mean error angle of ~20°, compared to the best case in EEG of ~40° previously reported [16]. For ECoG, power selected trials allow a mean error angle of ~30° in the best subject, compared to previously reported best results of ~60° [15]. Further post hoc analysis indicates that predictive performance is improved further by selecting trials by best fit to *past* data or by limiting predictions to initial blocks when the task was novel. Even so, the present performance surpasses previous attempts to predict *future* phase from within single time-series [14,15], from ERPs [9–12] and is comparable to our own, ‘best case’ event-related model, as follows.

**Table 1:** Prediction accuracy for each subject. List of subject-wise prediction accuracy at best time and frequency, for the best performing condition tested (varimax rotation, two eigenvectors, top 25% trials for *past* MLP). *n* is the number of trials (25% of total test set), except for the subject-wise mean, where *n* is the number of subjects. For the ‘Mean’ row, ‘SEM’ is the standard error of the subject means.

Subject	PLVerror	SEM	Frequency (Hz)	Time (ms)	n
MEG6	0.94	0.013	2.18	73.6	31
MEG13	0.88	0.02	6.96	57.6	39
MEG11	0.87	0.03	9.19	499.2	36
ECoG1	0.83	0.031	6.96	-195.31	37
MEG5	0.83	0.041	13.69	51.2	30
MEG2	0.81	0.045	9.35	-67.2	34
ECoG3	0.8	0.069	12.55	122.07	17
MEG16	0.79	0.038	11.12	-44.8	33
MEG4	0.78	0.041	2.42	499.2	36
MEG12	0.76	0.053	8.14	457.6	37
MEG7	0.71	0.068	2.04	-364.8	36
MEG19	0.7	0.062	2.04	-291.2	38
MEG10	0.69	0.064	2.64	-80	38
MEG15	0.69	0.069	13.93	0	36
MEG3	0.68	0.111	2.07	169.6	21
MEG9	0.66	0.103	10.74	-35.2	20
MEG14	0.62	0.074	2.26	-6.4	36
ECoG2	0.61	0.054	6.96	-136.72	75
MEG8	0.61	0.073	9.03	-156.8	33
MEG1	0.6	0.096	2.69	-208	33
MEG18	0.56	0.089	5.96	499.2	39
MEG20	0.53	0.088	9.51	185.6	35
MEG17	0.51	0.087	2.51	-384	35
Mean	0.72	0.025			23

### Local event-related model

**Fig 5:**
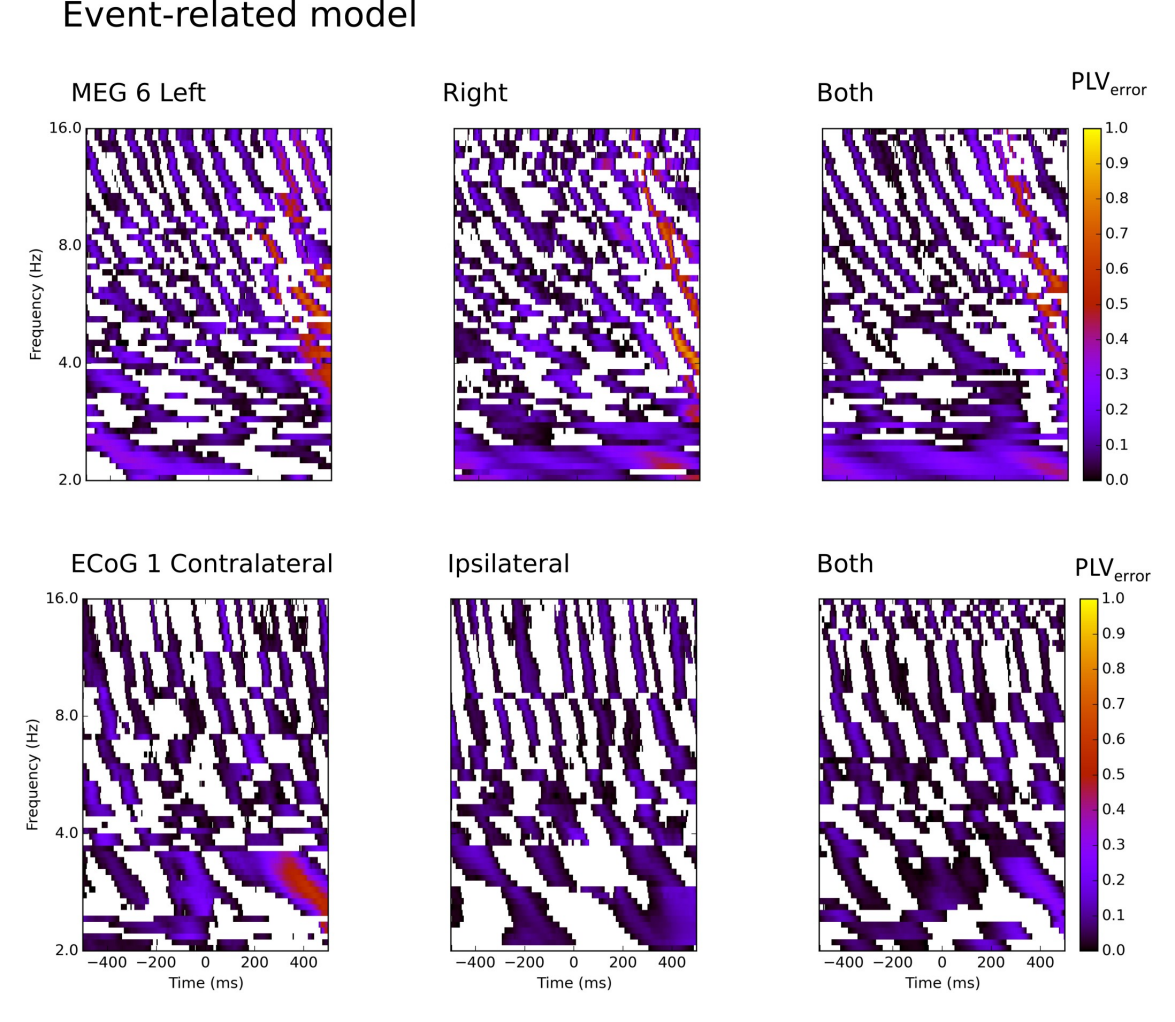
Event-related model results. PLV_error_ for the event-related model, the best predicted site at each frequency, chosen during model construction. Values are from the test data set for one MEG subject and one ECoG subject. The figure shows the prediction results for single hand motor activity (left, right or contralateral, ipsilateral) or for modelling results where the two trial-types were combined in the same analysis (both).

For comparison, we constructed localized event-related models [10,11] for each subject, condition (left or right hand movement, or both), frequency, time-in-trial and sensor. The model consisted of the trial-wise average phase-offset between *past* and *future* phase, and the best predicted site (for each subject, condition, frequency and time-in-trial) was found from the training data set. The models were tested on 50% of (previously unseen) trials. The PLV_error_ was higher than 0.9 in some subjects, at a broad range of frequencies, but only when the *past* phase was in the strictly event-related region of each trial. Example subjects’ results are shown in Fig 5 and S3 Fig. Some +250 to +500ms after button press, regions of good prediction can be found within narrow time-windows at each frequency. Outside of the event-related region, the performance was poor. Unlike the large-scale model, this event-related model required exact knowledge of the event-related structure of the task, since the mean phase offset over trials is calculated for each sample-within-trials. Consideration of Fig 5 suggests the event-related model predicts reverberatory-like activity following the relatively well-understood event-locked components of the motor task by one (ECoG) or two (MEG) cycles e.g. P3 following P2, hence the relative lateness of the components. This result is theoretically uninteresting, since the model could only predict events that follow known events. It is included here to provide a baseline. We do not include a grand-average plot, since the narrow bands of high PLV_error_ values tended not to always overlap from subject to subject, thereby giving misleadingly low values.

We assume that a self-initiated motor task comprises a best case for the kind of event-related model outlined here. Previous reports of event-related prediction, in cognitive paradigms, show much weaker results [7,9–12]. When we included both left and right button presses in the modelling—simulating a degree of task ambiguity—the performance of the event-related model dropped to the same level as the large-scale model. Statistical comparisons are reported at the end of the Results sections.

In short, the large-scale model performs well at all time samples, for at least some frequencies, not strongly dependent on the time-course of the task—here motor events followed by somatosensory and visual feedback. The event-related model only predicts phase events that follow already elicited, and known, phase events and its performance declines under conditions of task ambiguity.

### Tests of non-locality

We have established that the large-scale model out-performs the within time-series models which were the previous state-of-the art [14,15]. We also wanted to show that this predictive success was truly non-local and not due to inclusion of local information in the model. To this end we repeated the large-scale modelling procedure, but with the best predicted sites (found from the initial model’s training set) *excluded* from the *past* portion of each training vector during the second model construction phase. This meant that during the testing phase, the site to be predicted was also not included in the inputs to the model. One caveat: the varimax rotation modifies the weighting of each sensor on each eigenvector. This meant that the best predicted site sometimes differed for rotated components compared to the unrotated PCA. We therefore carried out the modelling reported in this section on unrotated PCA eigenvectors, in order to avoid introducing unnecessary inconsistency in the choice of best site.

**Fig 6:**
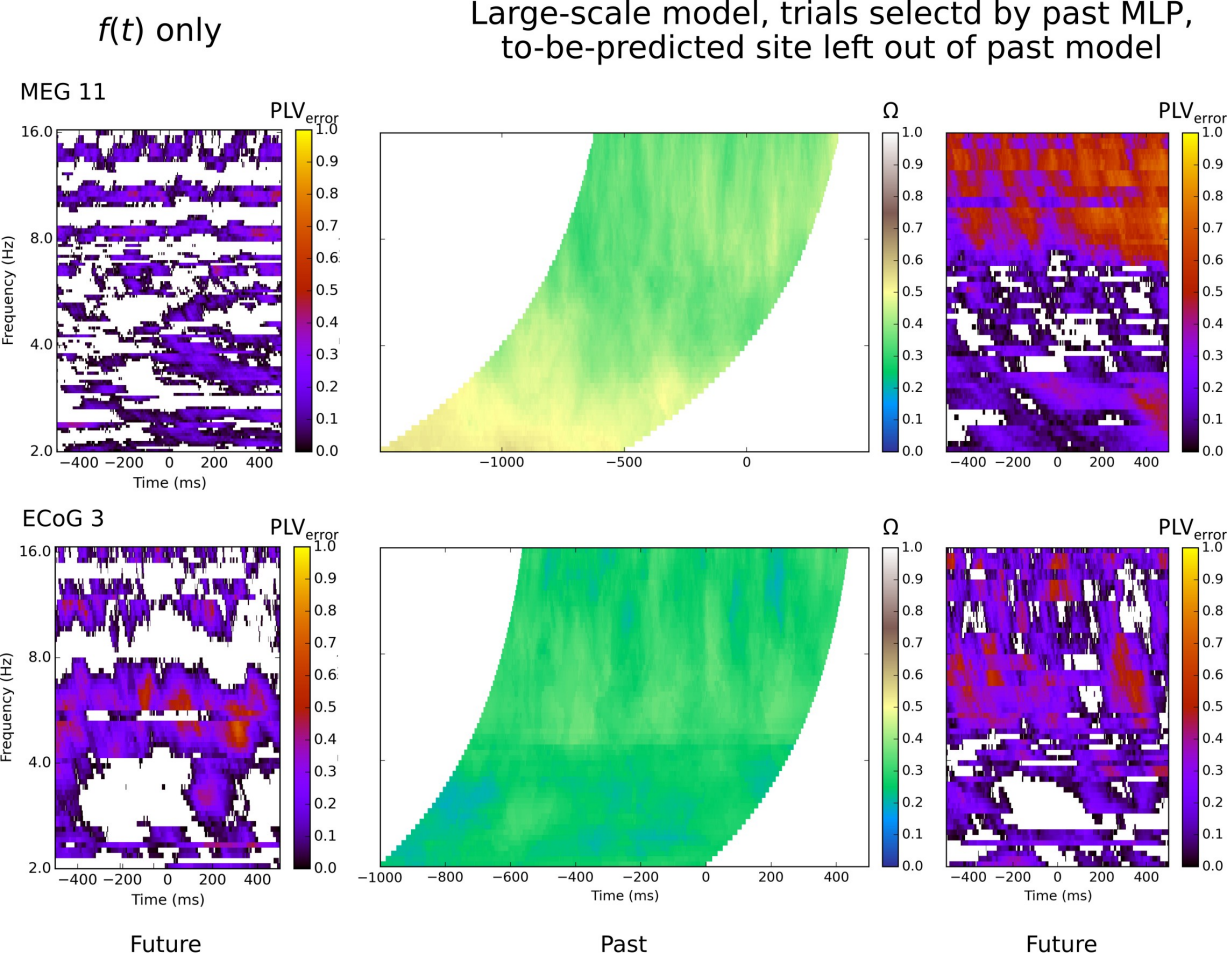
Trials selected by past MLP, model using single eigenvector, to-be-predicted site left out of past model. Only test trials where the past MLP was in the top quartile are included in this plot. Conventions are otherwise the same as for Fig 3.

Characteristic subjects’ results are included in Fig 6, and the best subjects show PLV>0.9 for 25% highest power trials. This result means that the predictive success of the large-scale model was not reliant on directly including data from the to-be-predicted site. MEG Subject 11 shows high prediction values late in the trial in the alpha and beta bands. ECoG Subject 3 shows best prediction accuracy late in the trial in the theta band. S4 Fig includes Subjects ECoG 1 and MEG 6, so the improvement provided by selecting trials (but excluding to-be-predicted site) can be compared to Fig 3. The first column in Fig 6 and S4 Fig shows the purely temporal Fourier prediction for the same subjects, also selected by past MLP. This method used only a single past site for future prediction; the same site that was excluded in the large-scale model without the to-be-predicted site. These plots demonstrate the unexpected result that the to-be-predicted site is not the best predictor for its own future activity; the large-scale dynamics provides a better predictor.

We do not include grand-average plots of these results as the inter-subject variation in times and frequencies meant the typical peak values were under-estimated by such representation. Additional *ad hoc* tests indicated moderate degradation of the prediction accuracy with up to a third of the neighbouring sites of the to-be-predicted site removed from the past inputs to the model.

**Fig 7:**
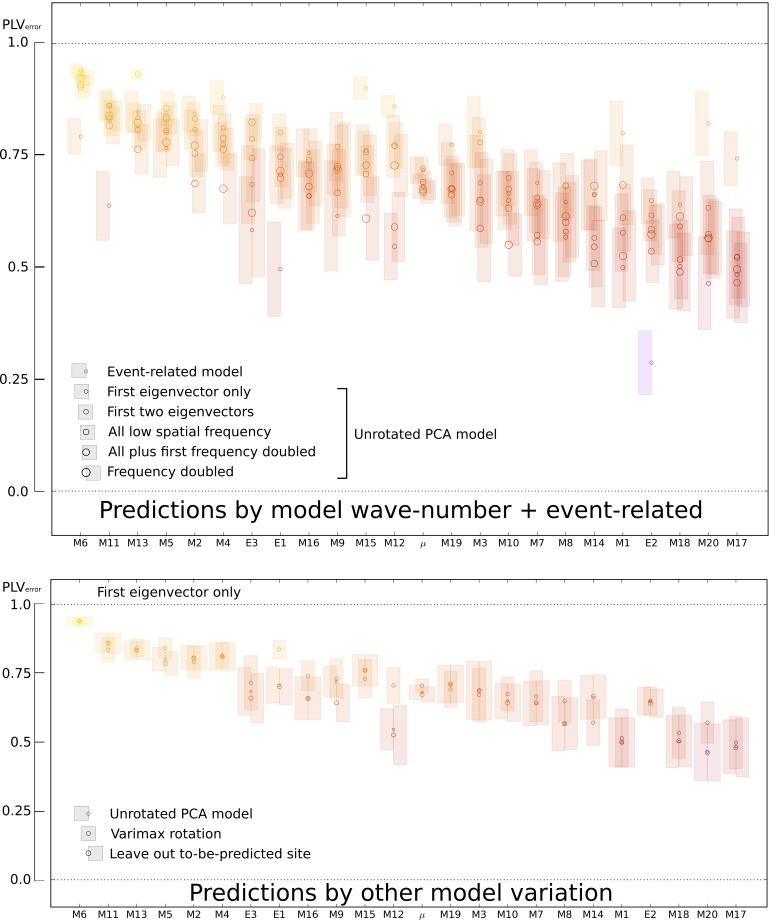
Mean error and SEM at best frequency and time for models with different wave-number and other model parameterizations. The results are shown for a quarter of the trials, those with the highest past MLP. Upper: results for the event-related model and large-scale TW model (unrotated PCA), across all subjects. Each subject’s PLV_error_, at the best time and frequency for that subject, are shown using circles. The event-related model and the models using only the first eigenvector are shown with smallest circles, and other configurations of eigenvectors are shown with successively larger circles, as indicated. Standard error of mean for the real part of the prediction error vector is shown in shaded regions (each subject’s *n* given in Table 1. The ‘*μ*’ column indicates the subject-wise mean of PLV_error_, and the subject-wise SEM of the PLV_error_ (n=23). MEG subjects are prefixed by ‘M’, ECoG subjects prefixed by ‘E’. Lower: results using the first eigenvector only, across all subjects. The plot compares unrotated PCA (smallest circles) with rotated PCA and the model with the to-be-predicted site removed, as indicated by the larger circles. Conventions are otherwise the same as for the upper figure.

As an additional check that the large-scale model was making use of the large-scale phase patterns to predict the phase value for the best-predicted site, we constructed PCA-based models from the spatial frequency-doubled eigenvectors (eigenvectors 4-6 or 5-8 for ECoG and MEG, respectively) for these same sites. The models performed less accurately than the low spatial frequency models (Fig 7). On average, the performance for three or four frequency-doubled eigenvectors was 0.06 (PLV_error_) less than when only the first, low spatial frequency eigenvector was used in the model.

Finally, we constructed models the same as the first models described, but with the inclusion of next eigenvector i.e. the first spatial-frequency doubled eigenvector. If this provided useful information to the large-scale model, it should have improved prediction performance. However, performance was degraded (see Fig 7). It was possible for performance to degrade, despite the availability more information, because we did not explicitly solve for the best predicting model. Using the present modelling procedures, inclusion of higher spatial frequency information has the effect of adding a noise term.

### Summary statistics

The chief parameterization findings for the large-scale model were summarized by way of a mixed linear model over all trials, with 1. the different configurations of model eigenvectors and 2. the types of models (unrotated PCA, varimax rotation, leave out to-be-predicted site) as the independent variables and trial-wise prediction accuracy as the dependent variable. The configurations of model eigenvectors in this analysis were, respectively, the first eigenvector only, the first two, all low spatial frequency (first three for ECoG, first four for MEG), all low frequency eigenvectors plus one (i.e. four for ECoG, five for MEG), and spatial frequency-doubled (three for ECoG, four for MEG). The all-low-plus-one and frequency-doubled conditions performed worse than the first eigenvector only (*p*<0.001 in both cases). The use of one, two or all low spatial frequency configurations did not differ statistically from each other. The rotated PCA performed better than both the unrotated PCA and the leave out to-be-predicted site condition (*p*<0.001). These latter two did not differ statistically from each other. The regression coefficients and standard errors are reported in S1 Table. In a separate mixed linear model, we also compared the large-scale model (one eigenvector, varimax rotation, top quartile *past* MLP) to the event-related model (condition ‘both’, top quartile *past* MLP) and the temporal Fourier model (top quartile past MLP). The former two models did not differ in mean performance (see S1 Table), but the temporal Fourier model performed less well.

While the event-related model performed better than the other models if analyzed with conditions separate (left vs. right motor), exactly the strength of the other models is that they do not require knowledge of the subjects’ event-related interactions with the experiment. For this reason we used condition ‘both’ in the reported analyses. *Ad hoc* analysis of a variety of other experimental paradigms (e.g. working memory task) confirms the intuition that the present self-initiated motor task approaches the best case for predicting future activity via the event-related model. With cognitive paradigms the performance of the event-related model drops to well below the Fourier-based models, consistent with published results [10–12]. In a similar vein, *ad hoc* testing suggests that the time-only Fourier model can surpass the predictive accuracy of the spatio-temporal model under conditions of highest cognitive loads (cf. 23). Exploring the boundary conditions [53] for when each of the three classes of models reported in this research surpasses the others in predictive accuracy may be an informative approach to understanding cortical function.

We conclude, therefore, that large-scale spatio-temporal models out-perform purely temporal models in the prediction of *future* phase and that the performance is similar to event-related models in the present setting.

## Discussion

### Ruling out alternative explanations

We were able to show that low spatial frequency TWs, measured over large regions of cortex, successfully predict future phase at localized sites of measurement. We showed this for MEG and ECoG data, which renders it unlikely that we are predicting trivial or non-signal components induced by volume conduction effects. Volume conduction effects cannot account for the spatiotemporal covariance the present models require for successful prediction, as such blurring is essentially instantaneous and therefore purely spatial [20,44,54].

The wide range of frequencies we were able to predict, from delta through to beta, also renders unlikely any other single, specific artefactual interpretation such as pulse artefact [55] or muscle activity [56]. The fact that we are *predicting* future activity provides the strongest argument against the artefactual character of the predictor signal. If the phase of activity recorded at a localized site represents real information used by the cortex [57], the inputs to the model cannot be more artefactual than competitor predictors which have substantially poorer performance.

The general emphasis on source localization in neuroscience is the reason for choosing to predict *local* activity from large-scale waves, however, our findings run counter to this emphasis. Our findings are not implausible in light of observations that ongoing [6,7] and low spatial frequency [3,28,43,44] activity dominate in the cortex. Large-scale cortical TWs can realistically be measured at the scalp or intracranially, because the relevant wavelengths are very long compared to the periodicity of the sulci and gyri (>10×). Additionally, large-scale neuromagnetic fields are constantly in motion [12]. This means smooth patterns of phase can be adequately sampled over time at the superficial gyri that contribute most to scalp measured signal.

### Properties of large-scale cortical waves

The models were constructed using PCA techniques, which in a number of measurement modalities have previously revealed consistent large-scale patterns of phase that explained more than half the variance in the phase data [12,23]. The novel techniques used in this research are akin to the empirical orthogonal functions utilized in meteorology and geophysics, although to our knowledge ours is the first model to incorporate explicit *past*-*future* representations as a means of modeling the future activity. The link to complex dynamical systems such as the study of fluid dynamics also suggests fruitful paths of exploration.

We showed that the predictive success of the model was only weakly dependent upon the number of eigenvectors used from the PCA. In fact, often the best predictions for the present data-sets generally used only the first, dominant eigenvector. This suggests a global mode of default cortical function associated with this predominant axis of flow. Task-related differences in the direction of flow that have been demonstrated to date [12,23,27,35] may constitute second order effects adding cognitive context to the first order flow.

We have previously noted that in MEG this dominant axis of flow is left-right in the frequency ranges delta to beta [12], while a number of studies have shown the predominant direction in EEG and ECoG to be anterior-posterior [1,23,25,33]. We have speculated that cortical activity is partly comprised of a global dynamical field that obeys the right-hand rule of electromagnetic fields [12], however this would constitute a distinct effect from localized electromagnetic dipoles assumed by mean field theory [58].

An issue of dispute in the study of traveling waves is the functional relevance of *large-scale* dynamics [59]. Here we showed that predictive success of large-scale waves was not dependent upon the presence of direct information from the to-be-predicted site on the input side of the model, and arises in both the whole cortex fields measured by MEG at the ~20cm cortical scale of ECoG. In addition, the best predictions arose from the long wavelength patterns: higher spatial frequencies did not fare as well. Together these findings argue for the functional relevance of large-scale interactions to cortical activity. An upcoming report will quantify the spatial tuning curves of past activity for local prediction accuracy.

The present results are timely, as there has recently been increased interest in the global organization of the cortex due to the availability of global imaging techniques for connectivity and myelination [60], function [61], plasticity [62] and gene expression [63]. A global cortical gradient of these various physiological and functional features has been proposed, spanning visual, auditory and somatosensory/motor functions, along with a unimodal/transmodal distinction which appears as gradient with twice the spatial frequency of the global gradient [64]. To this list we may include large-scale maps in the domain of neural dynamics [65].

Globally coherent TWs in the ECoG/MEG may reflect mechanisms involved in coordinating fast processes such as global activity binding [66], communication across large cortical distances [34,67], sequencing of activity [35], controlled access to memory processes [42], gating of information flow [30] and generalization of cognitive representations [65]. For such roles, global TWs must be able to interact with local cortical processing, but direct evidence for such a link is currently missing, to our knowledge. Accumulation of evidence showing when local activity is predicted better by previous large-scale activity than by the past local dynamics or event-related changes will allow a definitive assessment of the relationship between local and global cortical processes.

Where real-time prediction proves feasible, this will potentially provide for more flexible, ecological experiments, since tight event-related control is no longer be necessary to predict outcomes. The danger with event-related control of an organism is that experimenter can distort or even destroy the phenomena that was originally of interest [68]. If the intrinsic processes described by traveling waves are functionally important to environmental prediction and control, the organism should be given the opportunity to exercise them; something that is discounted in experimental paradigms where the next stimulus cannot be predicted.

### Implications for neuroscience experimentation and theory

From the present set of results we conclude that large-scale patterns—waves of wavelength 20 to 60cm—have a predictive relationship to future localized activity. The prediction success compares favorably to previous efforts using within time-series techniques—as high as 0.9+ PLV_error_ in the present research when trials were filtered by past power, compared to best values of 0.77 reported for within time-series measures [15], and compared to our own event-related models which were in the same range. Our best ECoG subject also improves upon previous attempts to predict future phase near the surface of the cortex: 0.82 compared to 0.50 PLV_error_ [14]. Like the previous research, we reported the results of the best performing predicted sensor for each subject. We used varimax rotation of PCA components to improve prediction accuracy. Current focus of research is on maximizing prediction accuracy at an arbitrarily nominated site by rotating the PCA solution.

Our result is surprising in view of the assumption of space-time separability common in standard methods such as ERPs; source localization methods; fMRI difference imaging. The implication of our result is that future activity is not merely a function of past activity in the same location and/or that in functionally coupled network locations [69,70]. That is, neural dynamics are not simply a spatial pattern of changing activations nor a temporal pattern at particular sites nor a combination of these two [13]; see Eq 1. The dynamics consist of moving activity, where the moving activity itself persists with sufficient coherence to accurately predict future activity at distal sites one or two cycles later at the frequency of interest. These results, unless and until bettered by alternative prediction approaches, suggest that the correct frame of reference for cortical activity is irreducibly spatiotemporal.

Our results, therefore, point to an essential shortcoming of analysis and modeling techniques in neuroscience that assume space-time separability. For example, pairwise coherence is effectively the average phase/amplitude relationship between two measurement sites (i.e. holding *x* constant, then aggregating over *t*; c.f. Eq 1). Attempts have been made to characterize large-scale cortical dynamics in these terms [36]. However, analysis of TWs [3,13] shows there are commonly several possible directions of wave activity, creating consistent but distinct sets of phase relationships between pairs of measurement sites, varying across trials but event-locked to the same task. This situation of multiple wave directions simply reflects phase relationships that are governed by multiple peaks in an activity function which are irreducibly dependent upon parametrization in space and time. This multiplicity of dynamics is directly analogous to the space-time separable view where there can be multiple temporal peaks or multiple spatial peaks. In the case of the multiple spatio-temporal peaks, coherence measures ‘wash out’ the real variation across these several modes of activity.

Beamformer techniques also assume space-time separability because of the key assumption that there is a underlying spatial pattern of signal sources, *G*(*x*), to be resolved from the measurements. Beamformer techniques aggregate (assess the variance of) spatial patterns of measured brain activity over trials to estimate the contribution of a specific brain region to the measured fields [71,72]. They represent an interesting methodological case because they explicitly try to solve the inverse problem by quantifying constructive and destructive interference patterns in the measured fields. We have previously argued that if the cortex’s fields are in motion, then the cross-trial interference pattern is an experimenter-created artifact that does not capture typical single-trial activity [3]. A strong rebuttal of this view would be to show that Beamformer techniques are able to predict future localized cortical activity, in real time, at greater accuracy than the presently described TW techniques.

Likewise explicit and therefore directly testable assumptions arise in the formulation of Independent Components Analysis. Here, the sources of the signals are assumed to be independent of each other (and to be some function of *x*), and the signals are aggregated over times in order to achieve blind separation of the signal sources.

Event-related potentials (ERPs) also assume space-time separability. In the present research, we contrasted prediction via waves with event-related models constructed with sample-by-sample parameterization. We included an analysis of left and right hand movements together, which resulted in a drop in prediction accuracy compared to when the conditions were analyzed separately. The drop reflects the absence of cross-trial consistency in the event-related signal, between the two conditions in this motor-related task. As a result of combining the two conditions, the cross trial aggregation fails to reflect some of the known structure in the signal. Yet this situation must be commonplace in cognitive neuroscience experiments, where we do not have access to the ground truth of which trials really are the same. We have previously argued [3,14] that event-related aggregation methods wash out non-summating signals in precisely the sense illustrated here. An even stronger illustration of signals that are washed out by summation are the patterns of phase prior to the event-related signal (e.g. between -100 and +100ms). These past patterns of phase must also be signal, since the traveling waves from these times predict the agreed-upon signal that arises between +100 and +400ms; yet they will be generally missed by methods that assume space-time separability.

In short, event-locked signals in cognitive neuroscience are often conflated with signals, *per se*. The present results demonstrates the presence of signal in the coherent patterns of waves preceding known event-locked signals by at least two cycles. Future research will examine the temporal range of future predictions.

Here we have used PCA and Fourier techniques to empirically estimate the intrinsically spatio-temporal frame of reference. Previous research on TWs has focused on single trial analysis of wave trajectories during different tasks [1,23,27] and subject states as well as between clinical groups [40,73–75]. The large-scale, single-trial waves described in this growing body of research cannot be explained by forward models of (a few) localized sources, due to the three observations about the dominant characteristics of cortical activity, outlined in the introduction. This kind of simple forward model would require the rest of the cortex to be silent at the single trial level, since by definition there can be no multi-trial aggregation over ‘noise’. But we know that most of the cortex is not silent at the single trial level. Any forward models of scalp measured activity must include high amplitude ongoing activity and high amplitude low spatial frequency activity in their account of wave-like patterns in single-trial EEG or MEG measurements [13]. Besides, forward models of scalp-observed wave patterns also need to account for the observed distribution of wave velocities, which increase with temporal frequency [76].

Previous work on traveling waves, like almost all neuroscience studies, has been correlational: showing that some pattern of brain activity is associated with experimental manipulations or other difference. The present results open the way for strong experimental prediction; namely that antecedent patterns of activity can be causally related to consequent effects. Here we invoke the distinction between generic, statistical-type ‘prediction’ common to psychology and neuroscience, and single event, point predictions [53]. An ambitious future goal would be to demonstrate causality by manipulating TWs directly via task-related changes [2], or possibly through transcranial magnetic stimulation [1,16,77].

For example, even better than showing, *post hoc*, that prior brain states are associated with seeing not seeing a near threshold stimulus [78–80], the present techniques may allow future states to be anticipated so that stimuli can be presented in real time to be seen or not seen. Likewise, BCI techniques for fast, real-time interactions are subject to high statistical variability [81–83]. The present techniques will reduce this variability which is not simply noise but, according to our analysis, arises due to treatment of the signal within time-series rather than as inherently spatio-temporal. Similarly, the present techniques may allow improvements in closed-loop transcranial magnetic stimulation techniques [1]. A pre-requisite to these applications, pursued in the present research, is to develop numerical methods for accurately predicting future cortical activity.

More generally, numerical techniques that allow prediction of future brain activity promise truly predictive experiments, and in turn stronger tests of theoretical predictions. Proposed mechanisms can be tested according to their predictive success on near future brain activity. Analysis methods such as Beamformer, ERPs and ICA should be compared to the present theoretical approach in terms of predictive success. Such predictive approaches will help control for the hidden variable interactions that plague correlational approaches. Finally, if future best-practice for predictive success substantiates the involvement of large scale spatio-temporal patterns of cortical phase, this would argue that these patterns are *sui generis* real and not an epiphenomenon [32,44].

## Methods

### MEG apparent motion task

Twenty human subjects (age range 22–36 years, mean age 27.1; 12 female) engaged in an audio-visual perceptual task, while their brain activity was recorded via MEG. All subjects were right-handed, had no audiological abnormalities, and had normal or corrected-to-normal vision. The task required subjects to choose the direction of motion of an audio-visual apparent-motion stimulus. Further details of the experiment can be found elsewhere [84]. Subjects were instructed to trigger the stimuli by pressing a button with either the left or right index finger, with random choice for each trial. In the blocks analyzed here, the audio-visual stimulus moved from the side indicated by the subject and then to the other side.

Neuromagnetic responses were recorded in a magnetically shielded booth using a 151-sensor whole-head gradiometer (CTF Systems Inc., Vancouver, Canada). Measurements were performed while subjects were seated. The MEG signals were sampled at 312.5 Hz. The time of the button press was designated as time zero in each trial. Future phase predictions were made for samples in each trial from −500 ms to +500 ms.

### ECoG finger movement task

Three patients suffering from intractable pharmacoresistant epilepsy took part in this study after having given their informed consent: ECOG1, female, aged 55 years, with a right fronto-polar focal cortical dysphasia (FCD); ECOG2, male, aged 26, years, with a left frontal FCD; ECOG3, male, aged 27 years, also with a left frontal FCD. All patients were strongly right-handed according to a modified Oldfield questionnaire and showed no clinical signs of pareses or other movement disorders. In ECOG1, a platinum electrode array (4 mm electrode diameter, 112 contacts, 7.1 mm inter-electrode distances) was subdurally implanted above the left fronto-parieto-temporal region for pre-neurosurgical diagnostics. In ECOG2 and ECOG3, 8x8 arrays (10 mm inter-electrode distances) were implanted above the left fronto-parietal cortex. Electrical stimulation was performed with the stimulator INOMED NS 60 (INOMED, Germany) to demarcate the eloquent brain areas. The intensity of stimulation was gradually increased up to 15 mA or to the induction of sensory and motor phenomena. All sites with arm or hand motor responses were located outside the ictal onset zone. A structural MRI data set with full head coverage was acquired on the day after electrode implantation using a T1 MPRAGE sequence.

Subjects were instructed to perform self-paced index finger flexions of either the left or the right hand with inter-movement intervals of at least 4 s. Here the behavioral task analyzed differed from the task reported in a previous study using the first subject [50]. The ECoG was recorded using a clinical AC EEG-System (IT-Med, Germany) digitized at 256 Hz (ECOG1) and 1024 Hz (ECOG2 and ECOG3) and band-pass filtered (0.032 Hz to 97 Hz). Further details of the subject recording can be found elsewhere [50]. ECoG data were re-referenced to average reference prior to data analysis. Onsets of index finger movement were determined in the electromyogram (EMG) of the M. flexor digitorum superficialis, pars indicis. Time zero in each trial was designated as the time of movement onset. Future phase predictions were made in samples in each trial from − 500 ms to + 500 ms, to incorporate most of the inter-movement interval. The movements contra-lateral and ipsi-lateral to implanted electrodes were both analyzed in the present study.

### Ethics statement

Written informed consent was obtained from all subjects prior to participation in the study. The MEG study was approved by the ethics committee of the University of Tübingen, Germany. The ECoG study was approved by the Freiberg University Clinic's ethics committee.

### Phase estimation

We let *T*, *S*, *L*, *F* denote—respectively—the sample times, measurement sites (ECoG electrodes or MEG sensors), trials and frequencies, as sequences of lengths *N_T_*, *N_S_*, *N_L_*, *N_F_*. The raw time-series signal is a 3-dimensional data set, *x*_*T*×*S*×*L*_. We used very short time-series wavelets to estimate the Fourier components, via either one cycle or two cycle Morlet wavelets (see S1 Methods), which have been shown previously to provide a good basis to characterize the short lasting wave events [23]. The Fourier components of the signal were estimated for a sequence of logarithmically-spaced center frequencies ranging from 2.0 to 16.0 Hz using the Morlet wavelets. We used 120 bands in order to assess a continuous gradient of frequency; in practice as few as 16 bands suffices to capture the frequency-based changes.

The Fourier components are denoted *X*_*T*×*S*×*L*×*F*_. From the Fourier components we define:

$$\Phi =e^{i\Psi }=X/\|X\|$$ (4)

which is the complex-valued phase represented by unit length complex numbers. Each has angle in the range −*π*≤Ψ<*π*. These quantities have the same indices *T*, *S*, *L*, *F*. In the above equation and for readability hereafter, some indices of *T*, *S*, *L* and *F* may be left implicit, depending on the context. We define mean log power (MLP) as

$$\mathrm{MLP}={\langle \log\left(\|X{\|}^{2}\right)\rangle }_{S}$$ (5)

that is, the mean over the sensors of the logarithm of the power. Angle brackets indicate the mean over the index, in this case the sensors. The MLP was used to select trials by power, since previous results [15,16] have suggested prediction is more accurate during epochs with high power.

### Estimate of wavelength

We estimate the spatial frequency distribution of the phases on the measurement array. This estimation can only be approximate in the present circumstances, since the array is irregular, is anyway a projection of the fields from the folded cortex, and the waves are an unquantified mixture of traveling planar, spiral and standing waves (c.f. [76]). Even so, some estimation of the dominant spatial frequencies of the field of phase measurements is useful. We partitioned the measurement array by computing the Delauney triangulation using the measurement sites as vertices. For each triangle, we computed the discrete spatial derivative of the phase as a vector of phase flow on the surface of the measurement array. The length of this vector gives a local approximation of the spatial frequency, and the distribution of the lengths over all triangles and samples give an indication of the typical spatial frequencies in the signal.

### Prediction model

The ECoG and MEG data of each subject were divided into a training set and a testing set, by assignment of odd numbered trials to the former and even trials to the latter. Alternative assignment schemes did not alter the results. The training set was used in model construction.

We followed previous efforts to capture large scale patterns of phase, using PCA to compute bases with desired properties of orthogonality, unit length, low rank, high variance explained and good generalizability [3,28]. The main difference in the present procedures is that we included a *past* and a *future* representation. In essence, we spatialized time for two discrete time-samples, by including the two offset samples in the PCA as part of the same case vector.

$${\overset{︷}{\Phi }}_{2T}=\Phi_{T-c}̑\Phi_{T+c}\equiv {\overset{︷}{\Phi }}_{past},{\overset{︷}{\Phi }}_{\mathrm{future}}$$ (6)

where ⁀ is concatenation, $\overbrace{\Phi }$ is the concatenated matrix of *past* of *future* phases, and the subscript *c* is an offset equal to half or one cycle of samples at the frequency *F* (ECoG and MEG, respectively). ${\overbrace{\Phi }}_{past}$ and ${\overbrace{\Phi }}_{future}$ can be read as simply elements of Φ organized in the correct time offsets.

We then constructed model bases that contained these representations of *past* phase, to be used in input side of the prediction procedure, and also representations of the *future* phase, to be used to make the predictions. Each input vector into the PCA therefore consisted of 2*N_S_* complex-valued phases. *Past* and *future* phase estimates are separated by one or two cycles, depending on the size of Morlet wavelet used. The *past* and *future* portions of the phase input vector were taken from abutting regions of the raw time series, so there was no overlap in the *past* and the *future* portions of the phase values in the input vector, nor in the raw time series they were estimated from. The ECoG gave better predictions using one cycle, while the MEG gave better predictions using two cycles, though the results were not strongly dependent on these choices. The phase of a sufficient number of samples into the *past* was calculated before-hand to enable *future* predictions within the ±500*ms* of the finger movement. See Fig 1 for a characterization of the interaction between frequency and time in the allocation of the correct *past* samples into the PCA.

For each subject and each frequency, the PCA was used to create a reduced-rank basis for the set of input vectors, from 1 to 3 (to 4 in the case of MEG) eigenvectors in size. The PCA was blind with respect to the event-related nature of the task, apart from the distinction between *past* and *future* samples, since we computed the eigenvectors once over all training trials and samples, as defined in Eq 6.

We tested a number of orthogonal rotation algorithms on the PCA output, and found that varimax rotation of the eigenvectors marginally improved the prediction results. Varimax rotation emphasizes the loading of individual variables on each eigenvector, and reduces the loading of the remaining variables. Since our goal was to predict the phase value at a single site, it made sense to emphasize the loading on individual measurement sites on the model bases.

The rotated eigenvectors were used as bases to constitute model versions of each sample, as follows. First, the *past* half of the input vector was regressed onto the *past* half of the bases, *W_past_* to estimate the model weights that best fit the *past* data.

$$\beta_{past}={\left(W_{past}^{†}W_{past}\right)}^{-1}\left(W_{past}^{†}{\overset{︷}{\Phi }}_{past}\right)$$ (7)

where † denotes the complex conjugate. The product of these weights and the *future* half of the bases, *W_future_*, gives the *future* model.

$$M_{future}=∠\;\left(\beta_{past}^{†}W_{future}\right)$$ (8)

where ∠ is the argument (angle of complex number). Since complex-valued PCA produces eigenvectors that are accurate up to a rotation of phase angle, we also calculated the model angle offset

$$\beta_{0}=\langle {\overset{︷}{\Phi }}_{future}/M_{future}\rangle_{TL}$$ (9)

Such that the angle corrected model is

$${\hat{M}}_{future}=M_{future}\beta_{0}$$ (10)

Note that *β*_0_ is a single complex number per subject per frequency per site; it does not provide any event-related information. We calculated the error between the *future* data phase and the *future* model phase for each measurement sensor, over all the times and training trials. The errors for the training data were given by

$$PLV_{train}=ℜ(\langle \frac{{\overset{︷}{\Phi }}_{future}}{{\hat{M}}_{future}}\rangle_{½TL)}$$ (11)

where R is the real part. From these errors we found the best predicted sensor for all trials and samples at a given frequency, *s*, for the training data set.

$$PLV_{s}=\begin{array}{c} \max\\ s\in S \end{array}PLV_{train}$$ (12)

Testing of the model was performed analogously to the second part of the model construction phase. The model components utilized are the first *n* eigenvectors from the PCA, *W_n_*, the index of the best predicted site, *s*, and the offset angle estimated for that site, *β*_*s*_∈*β*_0_. For each test sample, we compute the model test weights using the *past* half of the bases and the *past* test data vector, the same as Eq 7. These weights are applied to the *future* half of the bases to produce the *future* test model phases, as same in Eq 8. The test model *future* phase at the best training site is corrected for phase offset.

$${\hat{M}}_{s}=M_{s}\beta_{s}$$ (13)

*Error measures*: The error between the *future* model phase and the measured *future* phase at the best site is compared. The mean error is calculated as the real part of the phase-locking value (PLV) of the complex-valued errors found for the *future* test data. In practice, the PLV_error_ is calculated over all trials but for each sample, so we can see whether and how the prediction error changes in an event-related fashion.

$$PLV_{T}=ℜ(\langle \frac{{\overset{︷}{\Phi }}_{s}}{{\hat{M}}_{s}}\rangle_{½L)}$$ (14)

where ½*L* is the number of trials in the training or testing set. Because the number of cases in both the training sets and test sets was large (of the order 50,000 samples over half the trials) the error performance on the test set was very similar to the training set, diverging only after a few decimal places.

We trialed the effects of including all the long wavelength eigenvectors in the modelling, plus the first spatial frequency doubled eigenvector. In addition we trialed using only the frequency doubled eigenvectors in the model (eigenvectors 4-6 for ECoG, and 5-8 for MEG).

The large-scale wave model was compared to MLP values by use of Spearman Rho correlation coefficient. This was performed subject-wise, using *PLV_T_* at the best performing frequency and time-sample, and also within subjects using the equivalent trial-wise measure of error:

$$\varepsilon_{T\times L}=ℜ\left(\frac{{\overset{︷}{\Phi }}_{s}}{{\hat{M}}_{s}}\right)$$ (15)

*Event-related model and time-only Fourier model*: The event-related model is estimated separately for each subject, condition, frequency and time using the training data. It is simply the average phase offset between the trial averaged past phases and the trial averaged future phases, at each time *T* in the trial:

$${\bar{M}}_{future}=\frac{\langle {\overset{︷}{\Phi }}_{future}\rangle_{L}}{{\langle \overset{︷}{\Phi }}_{past}\rangle_{L}}$$ (16)

The estimated future phase, over all sites, is simply:

$${\bar{M}}_{future}={\overset{︷}{\Phi }}_{past}\times {\bar{M}}_{offset}$$ (17)

We find the best predicted site using Eq 11 (inserting ${\bar{M}}_{future}$ in place of ${\hat{M}}_{future}$) and 10, then apply the event-related model to the test data using Eq 17 and calculate errors using Eqs 14 and 15 (inserting ${\bar{M}}_{s}$ in place of ${\hat{M}}_{s}$). The time-only Fourier model is directly analogous to the event-related model, except that the average phase offset between *past* and *future* is calculated per frequency but over all times and trials.

## Supporting information

S1 Table Results of the mixed linear model. Upper table computes the model for PLV_error_ ~ Type + Bases, where Type is one of unrotated PCA, varimax rotated PCA and leave out to-be-predicted site. Bases are one eigenvector, two eigenvectors, all low spatial frequency eigenvectors, all low plus first spatial frequency doubled, and all spatial frequency doubled. All test trials were used in this analysis. The lower table compares the large-scale model (one eigenvector, varimax rotation) to the event-related model (condition ‘both’) and the time-only Fourier model. One quarter of the test trials were used in this second analysis, those that were in the top quartile for past test MLP. In both tables, the trial-wise data was collated for the best predicted site at the best time and frequency, for each separate measure. This means that a different site, time and frequency may be reported for the same subject over the various models and parameterizations.
(TXT)

S1 Fig Snapshots from phase prediction models for two MEG subjects and one ECoG subject. Conventions are the same as S1 and S2 videos.
(PDF)

S2 Fig PLV_error_ as a function of standard deviation of error angle. Curve is calculated assuming a Gaussian distribution of error angles about a mean of zero radians.
(PDF)

S3 Fig Event-related model results. PLV_error_ for the event-related model. Values are from the test data set. Conventions are otherwise the same as for Fig 5.
(PDF)

S4 Fig Trials selected by past MLP, model using single eigenvector, to-be-predicted site left out of past model. Only test trials where the past MLP was in the top quartile are included in this plot. Conventions are otherwise the same as for Fig 3.
(PDF)

S1 Video Animation of phase prediction, Subject MEG 6, 2.0Hz, first three trials, first eigenvector only, predicted site left out of past model. The movie is broken into five panels, showing the past phase (as a head map and on the complex plane), the past model phase, the model prediction of the future phase, the actual future phase, and the error between the model prediction and the future phase. The letters ‘A’,‘P’,‘I’,‘S’,‘L’,‘R’ indicate the position of the anterior-most (posterior-most etc.) recording site on either the head map or the unit circle of phase. The head maps move backwards and forwards to provide 3d perspective. For the past phase and future phase panels, head maps are shown in the centre of the panel, with unit circle of phase surrounding. The colours represent phase on both head and circle, and phase is reiterated as angular position on the unit phase circle. For this reason, purple is always at the top of circle, and green at the bottom. As phase on the head map changes, so individual sites, e.g. ‘A’, move around the unit phase circle. For the past model and model prediction, the model output is shown in complex values in the centre of the panel. The argument of the model i.e. the model angle, is shown in colours here and on the model head map (top left of these two panels). The site to be predicted is missing from the past model representation, on the bottom row of sensors, just to the right of the ‘L’ sensor. The site to be predicted is indicated with a black circle in both the model prediction panel and the future phase panel. When the prediction error is low, these two circles will traverse the same portions of their respective complex planes. The actual prediction error for the current movie frame is shown as a black ‘x’ in the last panel, on the unit circle of phase, here phase of the prediction error. When the prediction error is low, this cross will be at zero radians i.e. at 3 o'clock. Time within the present trial is indicated by the cross-hairs on the colour bar at the bottom of the error panel. The sample number within the present trial and the number of the present trial are shown in text inside the bottom half of the unit circle. The mean prediction error is shown on the coloured ‘time’ lines within the unit circle. The thin line with the dark cross-hairs indicates the mean prediction error over all trials at this time sample. The faint dotted line with the faint cross-hairs indicates the mean prediction error for the 25% of trials with highest mean log-power. The two estimates of mean prediction error are shown as text inside the top half of the unit circle. The error phase for all trials, at the present sample-within-trial, are indicated with coloured crosses on the unit circle. Cold colours indicate lower MLP, while hot colour indicate higher MLP.
(MP4)

S2 Video Animation of phase prediction, Subject ECoG 1, 6.7Hz, first three trials, first eigenvector only, predicted site left out of past model. Conventions are the same as for S1 video, except the letters ‘A1’,‘A8’,‘H1’,‘H8’ indicate the labels and positions of the corner recording sites of the ECoG array. The site to be predicted is missing from the past model representation, in position ‘B6’ i.e. one column in from the left, three rows down.
(MP4)

S3 Video Animation of phase prediction, Subject MEG 13, 2.14Hz, first three trials, first two eigenvectors, predicted site left out of past model. Conventions are the same as for S1 video. The site to be predicted is missing from the past model representation, and is on the right (far) side of the head map.
(MP4)

S4 Video Animation of phase prediction, Subject ECoG 1, 6.49Hz, first three trials, first three frequency doubled eigenvectors. Conventions are the same as for S2 video.
(MP4)

S1 Data Python routines implementing the spatio-temporal Fourier model. (PY)

S1 Methods Extended discussion of some of the issues behind using PCA to achieve the spatio-temporal Fourier analysis. (DOCX)
