# Supplementary material for: Large-scale cortical travelling waves predict localized future cortical signals
Source: PLoS Comput Biol. 2019 Nov 15;15(11):e1007316. doi: 10.1371/journal.pcbi.1007316 (PMC6894364; doi:10.1371/journal.pcbi.1007316)

MEG 11, 12.13Hz, first four eigenvectors, predicted site left out of past model

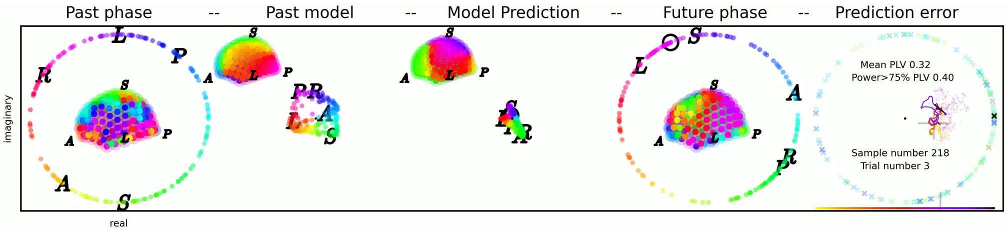

MEG 13, 2.14Hz, first two eigenvectors, predicted site left out of past model

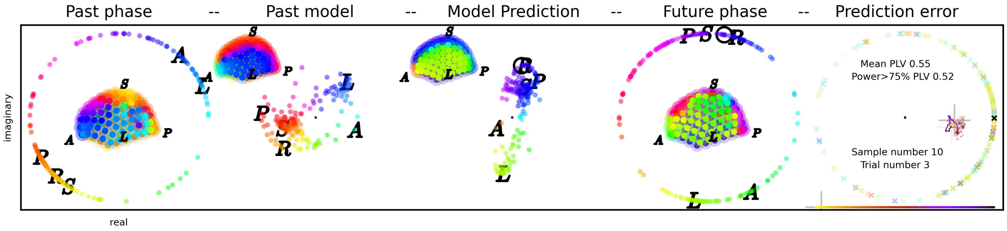

ECoG 1, 6.49Hz, three frequency doubled eigenvectors (i.e. eigenvectors 4 to 6)

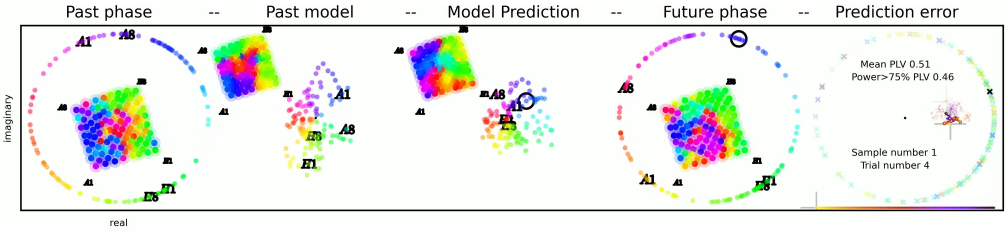

Supplement: S1 Fig — Conventions are the same as S1 and S2 videos. (PDF) [file pcbi.1007316.s002.pdf]
