# Supplementary figures and images for: Large-scale cortical travelling waves predict localized future cortical signals
Source: PLoS Comput Biol. 2019 Nov 15;15(11):e1007316. doi: 10.1371/journal.pcbi.1007316 (PMC6894364; doi:10.1371/journal.pcbi.1007316)

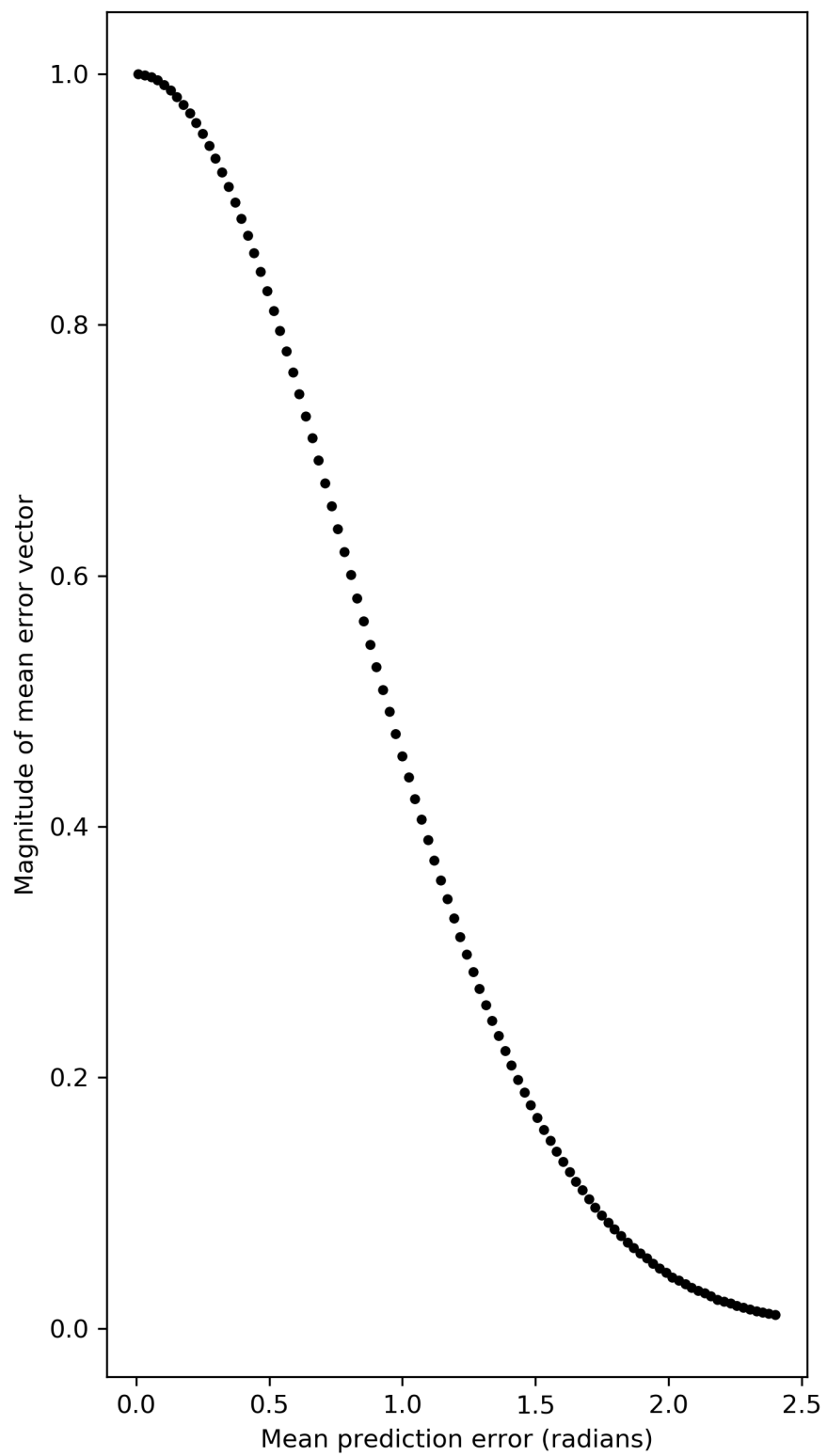

Supplement: S2 Fig — Curve is calculated assuming a Gaussian distribution of error angles about a mean of zero radians. (PDF) [file pcbi.1007316.s003.pdf]

## Event-related model

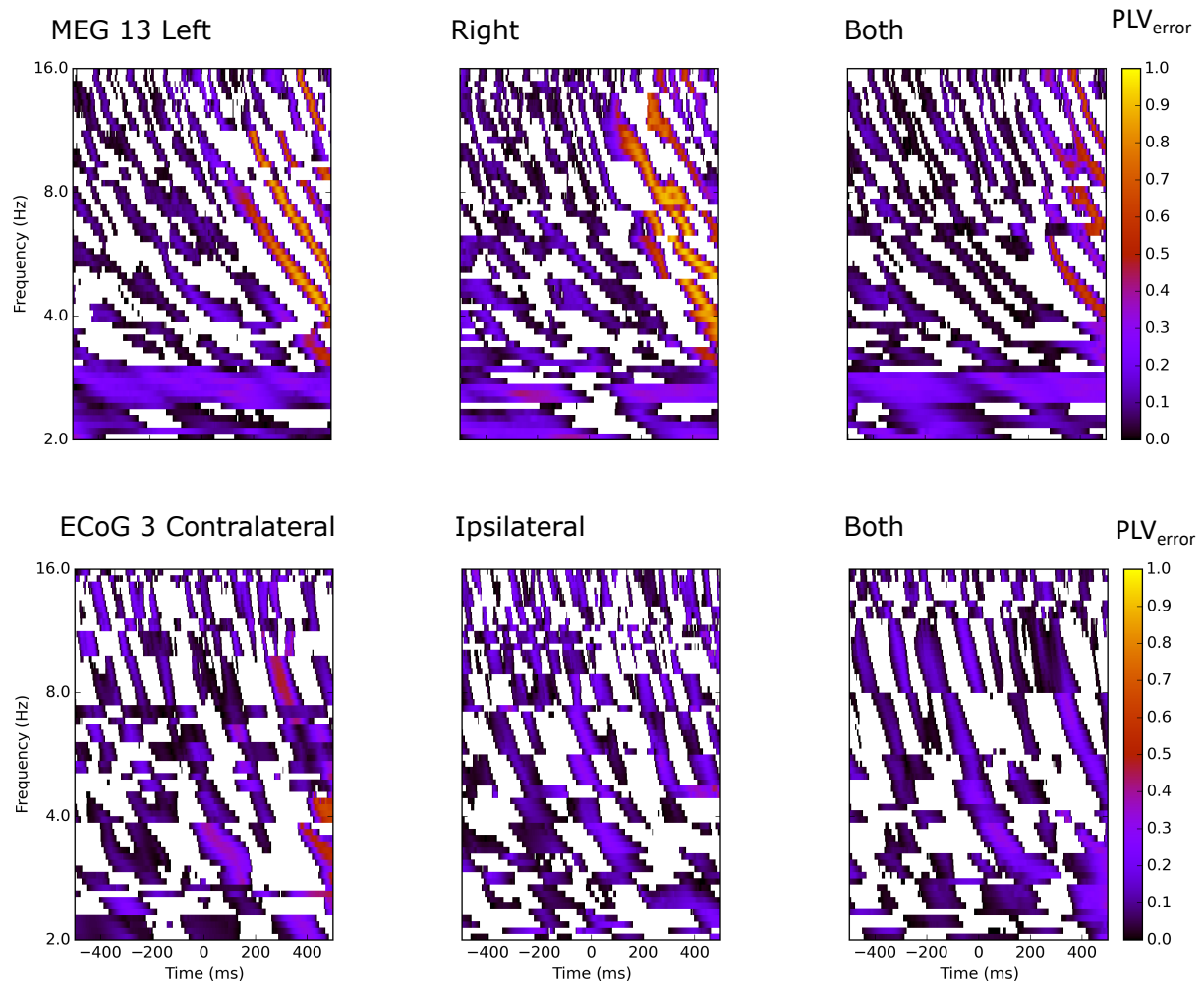

Supplement: S3 Fig — PLVerror for the event-related model. Values are from the test data set. Conventions are otherwise the same as for Fig 5. (PDF) [file pcbi.1007316.s004.pdf]

$f(t)$  only

Large-scale model, trials selected by past MLP,  
to-be-predicted site left out of past model

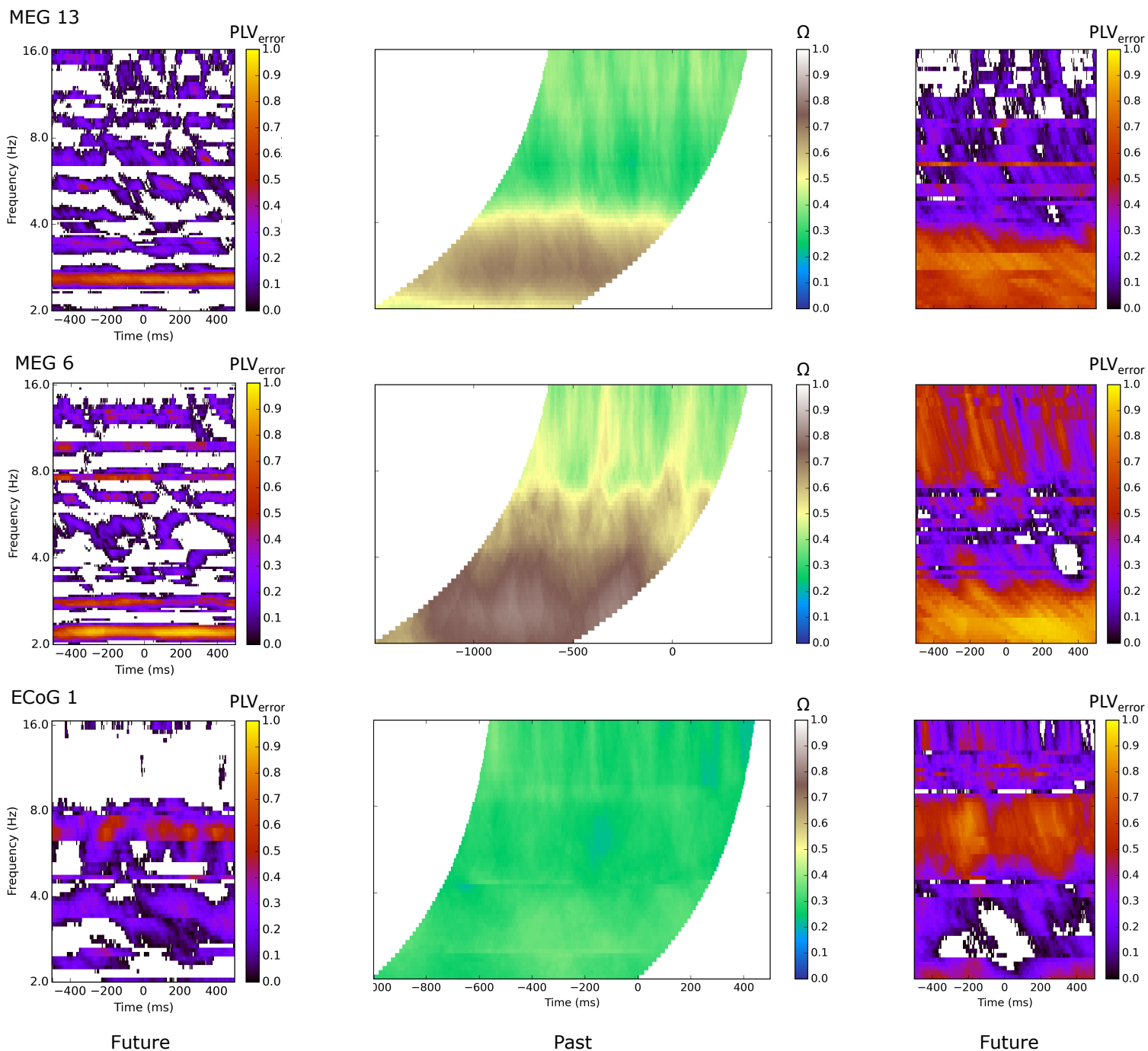

Supplement: S4 Fig — Only test trials where the past MLP was in the top quartile are included in this plot. Conventions are otherwise the same as for Fig 3. (PDF) [file pcbi.1007316.s005.pdf]
