# Supplementary material for: Large-scale cortical travelling waves predict localized future cortical signals
Source: PLoS Comput Biol. 2019 Nov 15;15(11):e1007316. doi: 10.1371/journal.pcbi.1007316 (PMC6894364; doi:10.1371/journal.pcbi.1007316)
Supplement: S1 Methods — (DOCX) [file pcbi.1007316.s011.docx]

*Supplementary Methods*

To analyze TWs we use Fourier-related methods. The Fourier transform over time and multiple spatial dimensions is defined as

$\underset{n}{\underbrace{\int_{-\infty}^{\infty} \cdots\int_{-\infty}^{\infty}}} {f\left( \mathbf{t} \right)e^{-2\pi i\mathbf{t}\cdot\mathbf{x}}d}^{n}\mathbf{t}$ (14)

for **t**, **x**∈R^n^. For the case of whole head MEG and ECoG arrays, the application of this transform is not straightforward, for a number of reasons. The measurement arrays are (1) *not regular* lattices, and the measurement sites tile only (2) *part* of the (3) *surface* of an of an object in three spatial dimensions which is only by (4) *approximation* a (5) *spheroid*, on which the (6) *wavelength* can sometimes be greater than the size of the array, and, (7) phase of the signal is calculated in complex coordinates, which on a spheroid, gives Fourier components in the form of *spherical harmonics*. Even though methods exist to address each of these problems, for example, least-squares spectral analysis for problem (1), addressing them jointly is complicated. Fortunately, the following observations simplify matters considerably.

Principal components analysis can be used to recover empirically-based Fourier components under appropriate conditions. For example, we can make an appropriately windowed 1/*f* noise time-series, where each window is a Gaussian spanning one cycle at *f*_max_. = 2π/*t*_range_. If we input many cases of these windowed data as input vectors into a scalar valued PCA, the PCA will recover as the first two eigenvectors Gaussian-windowed cos(*t*) and sin(*t*)—or phase-offset versions of these functions. The second pair of eigenvectors will approximate Gaussian windowed cos(2*t*) and sin(2*t*) and so on. This is because the PCA discovers as bases those functions that as weighted sums reproduce the original data, beginning with the factor that explains most of the variance. By definition this must be the basis functions of Fourier components (specifically approximations of Morlet wavelets), for the appropriately constructed example here.

We have previously demonstrated an analogous procedure using spatial vectors of *real-valued* EEG, ECoG or MEG phase—these are *spatially unwrapped* from the complex-valued phase. The vectors are input to a PCA, producing empirically-based spatial gradient functions (Alexander et al., 2006; 2013). The first three components (four in the case of MEG) are long-wavelength spatial gradients of real-valued phase that traverse the spatial array of sensor coordinates. Weighted sums of these gradients are able to reproduce global gradients oriented in any direction in the space of Cartesian coordinates defined by the array (see Supplementary Figures, Alexander et al., 2013). Later eigenvectors in the series have double the gradient slope of the first few eigenvectors, and so on.

In the present work, we make use of a similar approach, but beginning with the complex-valued spatial vector of phase. If we construct an array from many cases of spatial vectors of complex-valued phase from the MEG or ECoG, and use complex-valued PCA to find the eigenvectors, the first will be an approximate spiral wave with a spatial periodicity of unity over the size of the sensor array. Indeed, like the real-valued gradients of phase already discussed, the first three or four (MEG) eigenvectors will be global: here, approximate spiral waves oriented in different directions. Sometimes the bases look like mixtures of spiral waves and gradient waves. As weighted sums, this set of model waves can describe spiral waves turning about a polar axis which may be oriented in any arbitrary direction (See Supplementary Videos).

The amount of variance explained by the spiral wave eigenvectors is similar to that for spatial gradient eigenvectors—more than 50% of the variance in phase for any model wave with wavenumber equal to unity over the array (Alexander et al., 2006; 2016). Note that on a hemisphere or smaller spheroidal surface, most spiral models will fit the data approximately as well as gradient models e.g. a spiral wave with anterior-posterior poles vs. a gradient wave left to right. This means there is a large degree of overlap between either basis set or by bases that are mixtures of spiral and gradient waves in terms of the patterns of data they can model. The exception to this rule is the spiral wave with inferior-superior poles, which is approximately orthogonal to all three cardinal gradient directions in scalp coordinates. Like the case we described for empirically defined 1D Fourier series, and spatial gradients of real-valued phase, successive sets of eigenvectors constructed from complex-valued phase, after those with wavenumber of unity, are spatial frequency doubled and then spatial frequency quadrupled spiral-like waves.

Thus we are able to use the phase data in conjunction with PCA to discover the underlying spatial functions of phase that explain the largest amounts of variance in the single-trial phase.

Use of one cycle (or two) in the Morlet wavelets enables the phase and amplitude to be estimated from very short time windows, at the expense of frequency resolution ^73,74^. We have demonstrated that use of larger windows, such as in FFT or standard recommended size for Morlet wavelets (often 5 cycles), washes out the short-lived TWs (See Figure 7, Alexander et al., 2006).

Using 1-cycle Morlet wavelets may seem excessively short, even given the above consideration. However, three further considerations bear on this practice. First, substantial testing of 1, 2 or 3 three cycle Morlet wavelets does not drastically alter the patterns of waves that are found at different times and frequencies—this check has been undertaken on multiple data-sets and measurement modalities (D. Alexander, unpublished observation). Second, the present work constructs models using two adjacent estimates of phase—from abutting regions of the raw-time series. This means that the use of an adjacent pair of 1-cycle Morlet wavelets in the modelling has more similarities with standard use of 2-cycle Morlet wavelets, and the 2-cycle Morlets to 4. Third, the proof is in the pudding. Because we make use of separate training and test data sets, and because we predict future phase from past phase, any windowing artefact, aliasing noise or frequency blurring introduced by choice of methods will only decrease the accuracy of the results. The results suggest not.

We performed the PCA by computing the covariance matrix, *C*, on the normed array of case vectors, $\hat{\Phi}$, where the number of cases was *N_T_*×½*N_L_*.

$C_{S\times S}=\hat{\Phi}_{S\times½TL}^{\dagger}\hat{\Phi}_{S\times½TL}$ (15)

where † indicates the complex conjugate. Eigenvectors were then extracted using singular value decomposition. $C=W\hat{\Sigma}^{2}W^{\dagger}$ (16)

where *W* are the eigenvectors of Φ and $\hat{\Sigma}^{2}$ is the amount of variance explained for each eigenvector. PCA enables the covariance matrix to be numerically reorganized, in a lossless fashion, such that the first eigenvector lies along the axis that explains most of the variance in the data, and the second eigenvector lies along an orthogonal axis explaining the most residual variance, and so on up to the size of the covariance matrix. Since the eigenvectors are ordered by amount of variance explained, we can create a model basis using only the first few eigenvectors, and still explain most of the variance in the data. Because the model basis creates a ‘smoothed’ representation of the data (here by preferentially representing the low spatial frequency components of the phase patterns), the model has good generalizability.
